# Thioamination of Alkenes with Hypervalent Iodine Reagents

**DOI:** 10.1002/chem.201504636

**Published:** 2016-01-07

**Authors:** Pushpak Mizar, Rebecca Niebuhr, Matthew Hutchings, Umar Farooq, Thomas Wirth

**Affiliations:** a School of Chemistry, Cardiff UniversityPark Place, Main Building, Cardiff, CF10 3AT, UK), Fax: (+44) 29-2087-6968; b Department of Chemistry, COMSATS Institute of Information TechnologyAbbottabad, Pakistan

**Keywords:** addition, alkenes, amination, heterocycles, iodine

## Abstract

An efficient thioamination of alkenes mediated by iodine(III) reagents is described. The use of different sulfur nucleophiles allows the flexible synthesis of 1,2-aminothiols from alkenes. By employing chiral iodine(III) reagents, a stereoselective version of the thioamination protocol has also been developed.

Organic compounds containing sulfur and nitrogen heteroatoms are important building blocks for a board range of compounds with applications in biological, pharmaceutical, and material science. This continuous demand has encouraged the development of mild, safe, and highly selective procedures for their synthesis. Hypervalent iodine reagents are effective non-metallic reagents^1^,^2^ that have found many applications as highly selective oxidants^3^ and as electrophilic reagents^4^ for various reactions, including rearrangements^5^ and α-functionalizations of ketones.^6^ Taking into consideration our previous work on hypervalent iodine reagents and organocatalysis,^7^ we describe here a stereoselective and efficient procedure for the oxidative thioamination of alkenes.

Oxidative addition reactions to alkenes have been described in many publications, but methods involving the simultaneous addition of two different nucleophiles, such as nitrogen and sulfur, are rare. Denmark et al. recently developed efficient thioaminations based on sulfur electrophiles using chiral catalysts.^8^ The activation of the double bond with a hypervalent iodine reagent as electrophilic reagent is an alternative strategy, and a subsequent reaction with the first nucleophile leads to an intermediate, where the iodine(III) moiety is attached to a sp^3^-hybridized carbon atom (Scheme 1). The iodine(III) moiety is an excellent leaving group, several orders of magnitude more reactive than triflates or tosylates. This leaving group can be easily replaced with a second nucleophile to give 1,2-difunctionalized reaction products. The use of a second, external nucleophile, such as oxygen or nitrogen, has already been reported. By introducing sulfur nucleophiles as thiolates, direct thioaminations are possible.

**Scheme 1:**
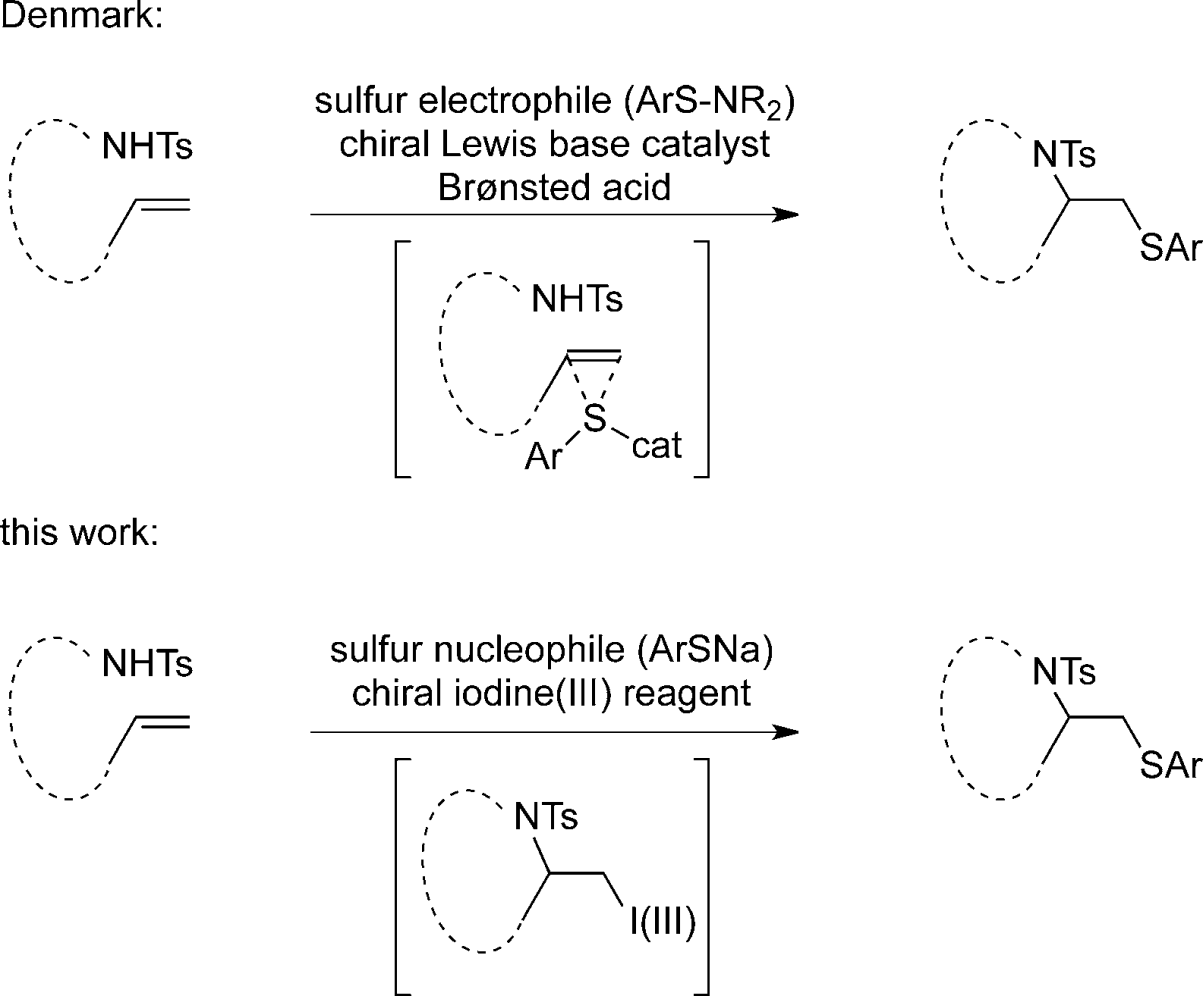
Strategies for thioaminations.

Direct thioaminations are very rare, as the alkene-activating reagent can directly react and oxidize the sulfur nucleophile. Thiourea moieties have been used as nucleophiles^9^ and, more recently, sulfilimines in reactions with alkynes.^10^ Even mild oxidants, such as iodine(III) reagents, can react with sulfur derivatives.^11^ Due to the thiophilic nature of hypervalent iodine reagents, different hypervalent reagents had to be screened, as the reagent should efficiently activate the alkene rather than react directly with the sulfur nucleophile. In order to evaluate hypervalent reagents and their reaction conditions for thioaminations, the reaction of 2-allyl aniline derivatives **1** with hypervalent iodine reagents under different reaction conditions in the presence of sodium thiophenolate was investigated as shown in Table 1.

**Table 1:** Reaction conditions for the thioamination of 1 using different iodine(III) reagents.

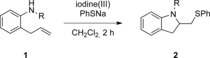				
Entry	Substrate	Iodine(III) reagent	Temperature [°C]	Yield [%]
1	**1 a** (R=Ts)	PhI(OAc)_2_	0	0^[a]^
2	**1 a** (R=Ts)	PhI(OH)OTs	0	5^[a]^
3	**1 a** (R=Ts)	PhI(OCOCF_3_)_2_	0	38^[a]^
4	**1 b** (R=Boc)	PhI(OCOCF_3_)_2_	0	0
5	**1 c** (R=Cbz)	PhI(OCOCF_3_)_2_	0	22
6	**1 a** (R=Ts)	PhI(OCOCF_3_)_2_	20	traces
7	**1 a** (R=Ts)	PhI(OCOCF_3_)_2_	−5	45
8	**1 a** (R=Ts)	PhI(OCOCF_3_)_2_	−20	72
9	**1 a** (R=Ts)	PhI(OCOCF_3_)_2_	−42	46
10	**1 a** (R=Ts)	PhI(OCOCF_3_)_2_	−75	32
11	**1 a** (R=Ts)	PhI(OCOCF_3_)_2_	−20	75^[b]^
12	**1 a** (R=Ts)	PhI(OCOCF_3_)_2_	−20	79^[c]^

[a] The use of other solvents (toluene, 2-propanol, and DMSO) did not result in any product formation. [b] Reaction time 1 h. [c] Reaction time 0.5 h.

Initially, different hypervalent iodine reagents were screened using 2-allyl-*N*-substituted anilines **1** as substrates at temperatures ranging from −75 to 20 °C in various solvents. With (diacetoxyiodo)benzene (Table 1, entry 1) and the Koser reagent (PhI(OH)OTs; entry 2), the reactions either did not proceed or the yields were very low, irrespective of the solvent used. However, the use of [bis(trifluoroacetoxy)iodo]benzene as iodine(III) reagent yielded the thioaminated product in moderate yield (entry 3) with dichloromethane as solvent. The low yield was due to side reactions taking place and hence the reaction temperature was decreased. Interestingly at −20 °C, the reaction proceeded best and within only 30 min the desired product was obtained in 79 % yield (entry 12). The nature of the protecting group also affected and influenced the reaction, the tosyl group led to highest yields. Similar products have already been obtained in two-step processes using substrates **1** in an aminoiodination/substitution sequence.^12^

With the optimized reaction conditions, the substrate scope of the reaction was explored. As summarized in Table 2, various *N*-(2-*a*llylphenyl)-4-methyl benzene sulfonamide derivatives were examined. In all cases the products were obtained in good yields (entries 1–3). The substrate scope was further extended successfully using pent-4-en-1-yl benzenesulfonamides (entries 4–6). In addition to the substrate scope, different thiolate nucleophiles were explored. As shown in Table 2, the products **13** and **14** (entries 7 and 8, respectively) were obtained in similar good yields.

**Table 2:** Substrate scope of the iodine(III)-mediated thioamination.

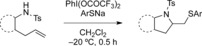				
Entry	Substrate	Nucleophile	Product	Yield [%]
	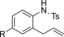		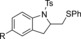	
1	**1 a** (R=H)	PhSNa	**2 a** (R=H)	79
2	**3** (R=Cl)	PhSNa	**4** (R=Cl)	75
3	**5** (R=OMe)	PhSNa	**6** (R=OMe)	70
	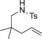		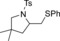	
4	**7**	PhSNa	**8**	60
	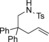		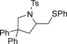	
5	**9**	PhSNa	**10**	71
	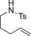		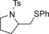	
6	**11**	PhSNa	**12**	56
			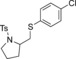	
7	**11**	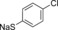	**13**	57
			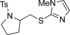	
8	**11**	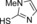	**14**	50

Encouraged by the success of the concomitant formation of the C−N and C−S bonds, we focused on the more challenging stereoselective synthesis of thioamination reaction products using chiral hypervalent iodine(III) reagents. Chiral hypervalent iodine(III) reagents have been very successfully used in stereoselective synthesis and received much attention.^13^ They have been used for the functionalizations of alkenes and also other substrates.^14^,^15^ For the development of a stereoselective thioamination reaction, different chiral iodine(III) reagents (Figure 1) were investigated. While the pyridine-substituted reagent **16**14h is superior to the conformationally less flexible reagent **15**^16^ (Table 3, entries 1 and 2), the highest selectivities were obtained with lactate-based hypervalent iodine reagents. Interestingly, only the C2-symmetrical reagents **18**^17^ led to good selectivities, whereas reagent **17**^18^ only delivered the racemic thioamination product (entry 3). With reagent **18 b**, the reaction temperature did neither influence yield nor selectivity (entries 6 and 7). A reaction temperature of −20 °C with 30 min reaction time was found to be ideal in generating the thioamination product **2 a** in 79 % *ee* (entry 5).

**Figure 1:**
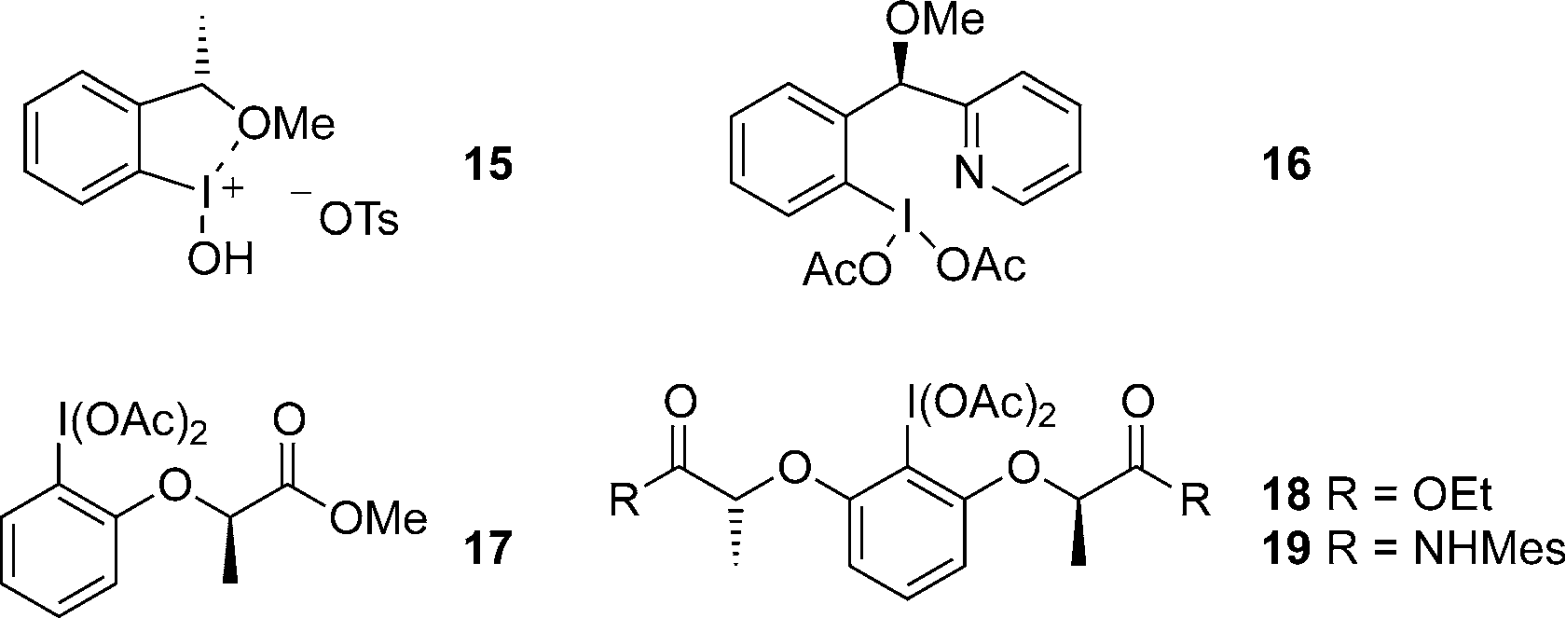
Selected chiral hypervalent iodine reagents.

**Table 3:** Stereoselective thioamination of 1 a with chiral iodine(III) reagents.

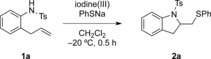			
Entry	Reagent	2 a: Yield [%]	2 a: *ee* [%] (absolute configuration)
1	**15**	51	34 (*S*)
2	**16**	54	69 (*R*)
3	**17**	32	0
4	**18 a**	48	52 (*R*)
5	**18 b**	68	79 (*R*)
6^[a]^	**18 b**	63	70 (*R*)
7^[b]^	**18 b**	65	71 (*R*)

[a] Reaction performed at −40 °C. [b] Reaction performed at −75 °C.

Different substrates were finally investigated in the stereoselective thioamination reaction using iodine(III) reagent **18 b** with the reaction conditions developed for substrate **1 a**. As shown in Table 4, some of the enantioselectivities obtained are promising with allylamine derivatives (entries 1 and 2) providing slightly higher selectivities than aliphatic pent-4-en-1-yl benzenesulfonamide derivatives (entries 3-5). The nucleophilicity of the sulfur nucleophile also influences the selectivity as can be seen by comparing the results in Table 4, entries 6 and 7. 1-Methyl-1*H*-imidazole-2-thiol provides a product with much lower enantiomeric excess than sodium thiophenolate.

**Table 4:** Substrates for the stereoselective iodine(III)-mediated thioamination.

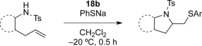				
Entry	Substrate	Product	Yield [%]	*ee* [%]
	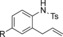	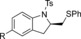		
1	**1 a** (R=H)	**2 a** (R=H)	68	79
2	**3** (R=Cl)	**4** (R=Cl)	50	74
3	**5** (R=OMe)	**6** (R=OMe)	53	70
	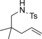	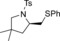		
4	**7**	**8**	47	60
	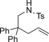	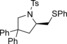		
5	**9**	**10**	57	61
	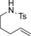	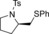		
6	**11**	**12**	42	55
	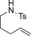	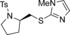		
7^[a]^	**11**	**14**	40	25

[a] Use of 1-methyl-1*H*-imidazole-2-thiol instead of sodium thiophenolate.

In summary, we have developed a flexible and efficient thioamination method of alkenes using iodine(III) reagents together with external sulfur nucleophiles. The protocol is straightforward, allowing the synthesis of a variety of pyrrolidine and indoline ring systems incorporating different thiol nucleophiles. The generation of 1,2-aminothiol derivatives from alkenes has been extended towards a stereoselective reaction by using lactate-based hypervalent iodine compounds.

## Experimental Section

**Cyclization of 1 a**: Into an oven-dried round-bottomed flask under nitrogen, a solution of *N*-(2-*a*llylphenyl)-4-methylbenzenesulfamide **1 a** (100 mg, 0.35 mmol) in dry CH_2_Cl_2_ (5 mL) was added dropwise to a suspension of [(bistrifluoroacetoxy)iodo]benzene (208 mg, 0.52 mmol) in dry CH_2_Cl_2_ (2 mL) at −20 °C. The reaction mixture was stirred for 15 min and treated carefully with the suspension of sodium benzenethiolate (46 mg, 0.35 mmol) in dry CH_2_Cl_2_ (3 mL). The reaction was stirred for further 15 min and quenched with saturated sodium thiosulfate solution (5 mL), diluted with water (5 mL), and extracted with CH_2_Cl_2_ (2×10 mL). The organic layers were combined, washed with brine (10 mL), and dried over MgSO_4_. They were filtered and the solvent was carefully removed under reduced pressure. The crude material was purified by column chromatography on silica gel using ethyl acetate/hexane (1:4).

## Supporting Information

As a service to our authors and readers, this journal provides supporting information supplied by the authors. Such materials are peer reviewed and may be re-organized for online delivery, but are not copy-edited or typeset. Technical support issues arising from supporting information (other than missing files) should be addressed to the authors.

## References

[1] Wirth T (2003). Hypervalent Iodine Chemistry, Vol. 224.

[2] Zhdankin VV (2014). Hypervalent Iodine Chemistry.

[3] Wirth T (2005). Angew. Chem. Int. Ed.

[4] Brown M, Farid U, Wirth T (2013). Synlett.

[5] Singh FV, Wirth T (2013). Synthesis.

[6] Merritt EA, Olofsson B (2011). Synthesis.

[7a] Richardson RD, Wirth T (2006). Angew. Chem. Int. Ed.

[7b] Singh FV, Wirth T (2014). Chem. Asian J.

[8a] Denmark SE, Hartmann E, Kornfilt DJP, Wang H (2014). Nat. Chem.

[8b] Denmark SE, Chi HM (2014). J. Am. Chem. Soc.

[9] Alleman S, Vogel P (1993). Synlett.

[10] Yoshida S, Yano T, Misawa Y, Sugimura Y, Igawa K, Shimizu S, Tomooka K, Hosoya T (2015). J. Am. Chem. Soc.

[11] Xia M, Chen Z-C (1997). Synth. Commun.

[12] Bovino MT, Chemler SR (2012). Angew. Chem. Int. Ed.

[13] For reviews, see:

[13a] Kumar R, Wirth T (2015). Top. Curr. Chem.

[13b] Berthiol F (2015). Synthesis.

[13c] Parra A, Reboredo S (2013). Chem. Eur. J.

[13d] Liang H, Ciufolini M (2011). Angew. Chem. Int. Ed.

[14a] Röben C, Souto JA, González Y, Lishchynskyi A, Muñiz K (2011). Angew. Chem. Int. Ed.

[14b] Farid U, Wirth T (2012). Angew. Chem. Int. Ed.

[14c] Singh FV, Rehbein J, Wirth T (2012). ChemistryOpen.

[14d] Souto JA, Martínez C, Velilla I, Muñiz K (2013). Angew. Chem. Int. Ed.

[14e] Kong W, Feige P, de Haro T, Nevado C (2013). Angew. Chem. Int. Ed.

[14f] Farid U, Malmedy F, Claveau R, Albers L, Wirth T (2013). Angew. Chem. Int. Ed.

[14g] Mizar P, Wirth T (2014). Angew. Chem. Int. Ed.

[14h] Mizar P, Laverny A, El-Sherbini M, Farid U, Brown M, Malmedy F, Wirth T (2014). Chem. Eur. J.

[14i] Mizar P, Burrelli A, Günther E, Söftje M, Farooq U, Wirth T (2014). Chem. Eur. J.

[15a] Uyanik M, Yasui T, Ishihara K (2010). Tetrahedron.

[15b] Fujita M, Yoshida Y, Miyata K, Wakisaka A, Sugimura T (2010). Angew. Chem. Int. Ed.

[15c] Fujita M, Wakita M, Sugimura T (2011). Chem. Commun.

[16a] Wirth T, Hirt UH (1997). Tetrahedron: Asymmetry.

[16b] Hirt UH, Spingler B, Wirth T (1998). J. Org. Chem.

[17] Uyanik M, Yasui T, Ishihara K (2010). Angew. Chem. Int. Ed.

[18] Fujita M, Okuno S, Lee HJ, Sugimura T, Okuyama T (2007). Tetrahedron Lett.

