# Supplementary material for: Thioamination of Alkenes with Hypervalent Iodine Reagents
Source: Chemistry. 2016 Jan 7;22(5):1614–7. doi: 10.1002/chem.201504636 (PMC4725223; doi:10.1002/chem.201504636)
Supplement: Supplementary file 1 — Supplementary [file chem0022-1614-sd1.pdf]

CHEMISTRY
A European Journal

Supporting Information

# Thioamination of Alkenes with Hypervalent Iodine Reagents

Pushpak Mizar,[a] Rebecca Niebuhr,[a] Matthew Hutchings,[a] Umar Farooq,[a, b] and Thomas Wirth*[a]

chem_201504636_sm_miscellaneous_information.pdf

**Supporting Information:**

Materials and Methods: All reactions were carried out under a nitrogen atmosphere in oven dried glassware with magnetic stirring using usual Schlenk technique. THF, toluene, diethyl ether and $CH_2Cl_2$ were purified and dried using standard methods. Triethylamine and diethylamine were distilled from sodium hydroxide. Reagents were purified prior to use unless otherwise stated. Purification of reaction products was carried out by flash chromatography using Fisher silica gel (35-70 mesh). NMR spectra were recorded on Bruker DPX 250, Bruker DPX 400, Bruker DPX 500, Bruker DPX 600, or Oxford 300. $^1H$ NMR spectra were measured at 250, 300, 400 and 500 MHz. $^{13}C$ NMR spectra were measured at 63, 100, 126 and 150 MHz using $CDCl_3$, or DMSO-$d_6$ as a solvent and internal reference. Coupling constants J are given in Hz. Multiplicity as follows: s = singlet, d = doublet, t = triplet, q = quartet, m = multiplet, br = broad signal. Mass spectrometric data were obtained on a Varian 1200 Quadrupole Mass Spectrometer and Micromass Quadro II Spectrometer.

*Typical procedure for the synthesis of 2-allylanilines* [1]

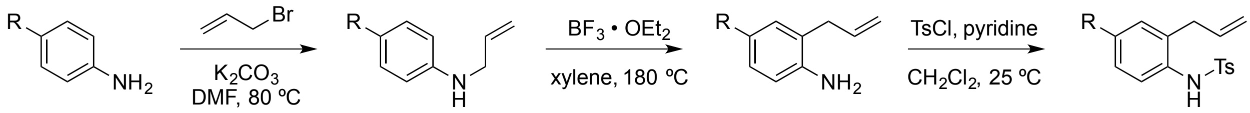


Allylbromide (1.40 mL, 16.24 mmol) was added dropwise to a solution of commercially available aniline (16.24 mmol) [aniline: 1.51 g, 4-methoxyaniline: 2.00 g, 4-chloroaniline: 2.06 g] and $K_2CO_3$ (5.39 g, 38.97 mmol) in DMF (37 mL). The solution was heated to 80 ºC and stirred at this temperature overnight. The reaction mixture was then filtered, washed with water (3 x 20 mL) and extracted with EtOAc (2 x 15 mL). The combined organic extracts were washed with brine (30 mL), dried over $Na_2SO_4$ and concentrated in vacuo. The crude product was purified by column chromatography ($CH_2Cl_2$) to afford the *N*-allyl aniline as yellowish oil. Next, $BF_3$•$OEt_2$ (0.93 mL, 7.36 mmol) was added to a solution of the *N*-allyl aniline (7.36 mmol) in xylene (4 mL). The mixture was heated to 180 ºC in a sealed tube and stirred at this temperature for 2 h. After cooling, the reaction mixture was poured into 10% aq NaOH (10 mL), and extracted with EtOAc (3 x 15 mL). The combined organic extracts were washed with brine (10 mL), dried over $Na_2SO_4$, and concentrated in vacuo. The crude product was purified by column chromatography ($CH_2Cl_2$/EtOAc, 4:1) to yield the 2-allyl aniline as colourless oil. The $^1H$ and $^{13}C$ NMR were in agreement with the reported data.[6] The anilines were protected with the tosyl group using the procedure described above.

***N*-(2-Allylphenyl)-4-methylbenzenesulfonamide (1a)**

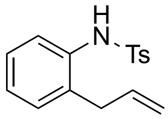


Tosylation yield: 0.45 g (1.56 mmol, 90%). $^{1}$H NMR ($CDCl_3$, 400 MHz): δ = 7.51 (d, J = 8 Hz, 2H), 7.35 (dd, J = 1, 8 Hz, 1H), 7.13-7.18 (m, 3H), 6.96-7.08 (m, 2H), 6.43 (br, 1H), 5.67-5.74 (m, 1H), 5.03 (dd, J = 1.5, 10 Hz, 1H), 4.85 (dd, J = 2, 11 Hz, 1H), 2.92 (d, J = 4Hz, 2H), 2.33 (s, 3H) ppm; Spectral data are in agreement with reported data.[6]

***tert*-Butyl (2-allylphenyl)carbamate (1b)**

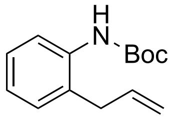


Boc-protection yield: 67%. $^{1}$H NMR (300 MHz, $CDCl_3$): δ = 7.79 (d, *J* = 8.7 Hz, 1H), 7.25-7.23 (m, 1H), 7.15 (d, *J* = 7.5 Hz, 1H), 7.05 (t, *J* = 7.2 Hz, 1H), 6.44 (br, 1H), 6.03-5.90 (m, 1H), 5.16 (dd, *J* = 1.5, 9.0 Hz, 1H), 5.07 (dd, *J* = 1.5, 16.8 Hz, 1H), 3.37 (d, *J* = 5.7 Hz, 1H), 1.53 (s, 9H) ppm; Spectral data are in agreement with reported data.[2]

**Benzyl (2-allylphenyl)carbamate (1c)**

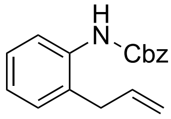


Carboxybenzylation yield: 62%. $^{1}$H NMR (300 MHz, $CDCl_3$): δ = 7.82 (d, *J* = 8.0 Hz, 1H), 7.42-7.23 (m, 6H), 7.19 (dd, *J* = 2.0, 7.6 Hz, 1H), 7.09 (td, *J* = 1.2, 7.6 Hz, 1H), 6.61 (br s, 1H), 5.96 (m, 1H), 5.22 (s, 2H), 5.15 (m, 1H), 5.07 (m, 1H), 3.37 (m, 2H) ppm; Spectral data are in agreement with reported data.[3]

***N*-(2-Allyl-4-chlorophenyl)-4-methylbenzenesulfonamide (3)**

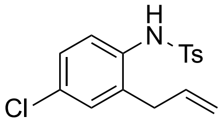


Tosylation Yield: 1.4 g (4.3 mmol, 87%). (Overall yield: 60%). $^{1}$H NMR ($CDCl_3$, 400 MHz): δ = 7.61 (d, J = 8Hz, 2H), 7.38 (d, 8 Hz, 1H), 7.25 (d, J = 8Hz, 2H), 7.21 (dd, J = 2, 8 Hz, 1H), 7.10 (d, J = 2Hz, 1H), 6.47 (br, 1H), 5.73-5.81 (m, 1H), 5.17 (dd, J = 1.6, 12 Hz, 1H), 4.96 (dd, J = 1.5, 17 Hz, 1H), 2.98 (d, 6Hz, 2H), 2.45 (s, 3H); Spectral data are in agreement with reported data.[6]

**N-(2-Allyl-4-methoxyphenyl)-4-methylbenzenesulfonamide (5)**

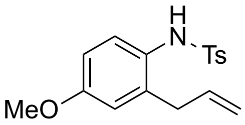

Tosylation Yield: 1.2 g (3.8 mmol, 89%) (Overall yield: 65%). $^1$H NMR ($CDCl_3$, 400 MHz): δ = 7.58 (d, J = 8Hz, 2H), 7.26 (d, J = 8Hz, 2H), 6.77 (m, 2H), 6.65(s, 1H), 6.25 (br, 1H), 5.74-5.81 (m, 1H), 5.14 (dd, J = 2, 12 Hz, 1H), 4.98 (dd, J = 2, 13 Hz, 1H), 3.81 (s, 3H), 2.94 (d, J = 5 Hz, 2H), 2.45 (s, 3H) ppm; Spectral data are in agreement with reported data.[6]

*Procedure for Cyclisation:*

Into an oven-dried round-bottom flask under nitrogen a solution of *N*-(2-allylphenyl)-4-methylbenzenesulfamide **1a** (100 mg, 0.35 mmol) in dry $CH_2Cl_2$ (5 mL) was added dropwise to a suspension of [(bistrifluoroacetoxy)iodo]benzene (208 mg, 0.52 mmol) in dry $CH_2Cl_2$ (2 mL) at –20 °C. The reaction mixture was stirred for 15 min and treated carefully with suspension of sodium benzenethiolate (46 mg, 0.35 mmol) in dry $CH_2Cl_2$ (3 mL). The reaction was stirred for further 15 min and quenched with saturated sodium thiosulfate solution (5 mL), diluted with water (5 mL) and extracted with $CH_2Cl_2$ (2 x 10 mL). The organic layers were combined, washed with brine (10 mL) and dried over $MgSO_4$, filtered and the solvent was carefully removed under reduced pressure. The crude material was purified by column chromatography on silica gel using ethyl acetate/hexane (20%).

**2-((Phenylthio)methyl)-1-tosylindoline (2a)**

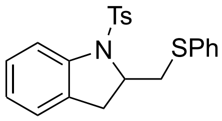

$^1$H NMR (400 MHz, $CDCl_3$): δ = 7.67 (d, *J* = 8 Hz, 1H), 7.59 (d, *J* = 8 Hz, 2H), 7.25 – 7.15 (m, 6H), 7.09 – 7.00 (m, 4H), 4.40 – 4.31 (m, 1H), 3.70 (dd, *J* = 10, 3 Hz, 1H), 3.30 (t, *J* = 10 Hz, 1H), 2.98 (dd, *J* = 17, 9 Hz, 1H) 2.34 (dd, *J* = 17, 3 Hz, 1H), 2.37 (s, 3H) ppm; $^{13}$C NMR (100 MHz, $CDCl_3$): δ = 144.4, 141.3, 136.1, 134.7, 130.5, 129.9, 129.5, 128.9, 128.1, 127.2, 125.4, 125.2, 125.0, 116.9, 62.7, 34.9, 129.4, 21.7 ppm; HRMS (ESI): calc. for $C_{22}H_{22}NO_2S_2$ $[M+H]^+$: 396.1014, found: 396.1022.

**Benzyl 2-((phenylthio)methyl)indoline-1-carboxylate (2c)**

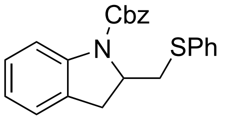


$^1$H NMR (400 MHz, $CDCl_3$): δ = 7.86 (br s, 1H), 7.47–7.31 (m, 10H), 7.21 (br s, 1H), 7.17 (d, $J$ = 7.5 Hz, 1H), 7.00 (t, $J$ = 7.5 Hz, 1H), 5.32 (s, 2H), 4.93 (br s, 1H), 3.41 (dd, $J$ = 16.5, 4.5 Hz, 1H), 3.27 (br s, 1H), 2.93 (m, 2H) ppm.

**5-Chloro-2-((phenylthio)methyl)-1-tosylindoline (4)**

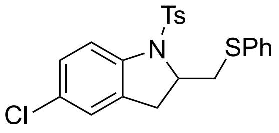


$^1$H NMR (400 MHz, $CDCl_3$): δ = 7.59 (dd, $J$ = 4.8, 8.4 Hz, 1H), 7.44 (d, $J$ = 7.6 Hz, 2H), 7.39-7.34 (m, 4H), 7.27-7.23 (m, 1H), 7.11 (d, $J$ = 8.0 Hz, 2H), 6.91 (td, $J$ = 2.4, 9.2 Hz, 1H), 6.74 (dd, $J$ = 2.8, 8.0 Hz, 1H), 4.30-4.22 (m, 1H), 3.67 (dd, $J$ = 3.6, 13.6 Hz, 1H), 2.92-2.73 (m, 3H), 2.34 (s, 3H) ppm; $^{13}$C NMR (100 MHz, $CDCl_3$): δ = 160.4, 144.1, 137.2, 134.7, 134.2, 133.3, 129.6, 129.1, 126.9, 126.3, 118.2, 114.5, 112.4, 61.2, 38.5, 33.2, 21.5 ppm; IR (neat): 3058, 2923, 1599, 1482, 1356, 1166, 1090, 1026, 935, 814 cm$^{-1}$; HRMS (ESI): calc. for $C_{22}H_{21}ClNO_2S_2$ $[M+H]^+$: 430.0624, found: 430.0618.

**5-Methoxy-2-((phenylthio)methyl)-1-tosylindoline (6)**

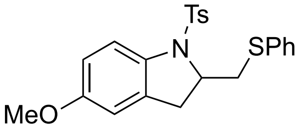


$^1$H NMR (400 MHz, $CDCl_3$): δ = 7.53 (dd, $J$ = 17, 9 Hz, 3H), 7.21-7.27 (m, 5H), 7.17 (d, $J$ = 8 Hz, 2H), 6.77 (dd, $J$ = 9, 3 Hz, 1H), 6.60 (d, $J$ = 3 Hz, 1H), 4.36 – 4.28 (m, 1H), 3.78 (s, 3H), 3.64 (dd, $J$ = 10, 4 Hz, 1H), 3.26 (t, $J$ = 10 Hz, 1H), 2.84 – 2.70 (m, 2H), 2.39 (s, 3H) ppm; $^{13}$C NMR (100 MHz, $CDCl_3$): δ = 157.8, 144.3, 135.7, 134.7, 134.4, 132.5, 129.8, 129.6, 128.3, 127.3, 125.7, 118.3, 113.3, 111.0, 63.0, 55.7, 39.2, 35.0, 21.8 ppm; HRMS (ESI): calc. for $C_{23}H_{24}NO_3S_2$ $[M+H]^+$: 426.1119, found: 426.1126.

*Procedure for the synthesis of starting materials **S1** and **S2***

Into an oven-dried round-bottom flask under nitrogen, sodium hydride (60% in oil, 3.52 g, 91.6 mmol) and dimethylformamide (10.3 mL) was added at 0 °C. Diphenylacetonitrile (2.0 g, 10.35 mmol) [or isobutyronitrile (0.71 g, 10.35 mmol)] was added portionswise over 10 min and stirred

45 min at 0 °C. The resulting suspension was treated dropwise with allylbromide (1.57 mL, 18 mmol), warmed up to room temperature and stirred for 16 h. The resulting solution was cooled to 0 °C and quenched by adding water (20 mL). The mixture was diluted 1:1 with diethyl ether washed with water (3 x 10 mL) and brine (1 x 10 mL). The aqueous layers were extracted with $CH_2Cl_2$ (1 x 15 mL). The combined organic layers were dried with $MgSO_4$ and filtered and concentrated under reduced pressure. The crude material was purified by column chromatography on silica gel using ethyl acetate/hexane (1:5).

**2,2-Diphenyl-pent-4-enenitrile (S1)**

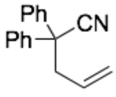


Yield: 3.3 g (14.1 mmol, 70 %); yellow oil.

$^1$H NMR (300 MHz, $CDCl_3$): δ = 7.15-7.30 (m, 10H), 5.56-5.65 (m, 1H), 5.04-5.14 (m, 2H), 3.02-3.04 (d, $J$ = 7 Hz, 2H) ppm; Spectral data are in agreement with literature.[4]

**2,2-Dimethyl-pent-4-enenitrile (S2)**

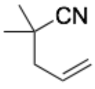


Yield: 1.8 g (16.4 mmol, 62%); colourless oil.

$^1$H NMR (300 MHz, $CDCl_3$): δ = 5.78-5.95 (m, 1H), 5.25-5.15 (m, 2H), 2.27-2.30 (d, $J$ = 7 Hz, 2H), 1.34 (s, 6H) ppm; Spectral data are in agreement with literature.[4]

*General method of reduction using lithium aluminium hydride*

Into an oven-dried round-bottom flask under nitrogen a solution of compound **S1** (3.3 g, 15 mmol) [or compound **S2** (1.63 g, 15 mmol)] in $Et_2O$ (100 mL) was added dropwise to a suspension of $LiAlH_4$ (1.2 g, 32 mmol) in $Et_2O$ (250 mL) at 0 °C, warmed up to room temperature and stirred for 16 h. The reaction mixture was cooled to 0 °C and treated carefully with 20% aq. NaOH (5 mL), then diluted with $Et_2O$ (50 mL) and the precipitation salts were filtered. The precipitation was washed with $Et_2O$. The organic layers were combined, washed with brine (3 x 10 mL) and dried over $MgSO_4$, filtered and the solvent was carefully removed under reduced pressure. The crude material was purified by column chromatography on silica gel using ethyl acetate/hexane (1:5).

**2,2-Diphenyl-pent-4-enylamine (S3)**

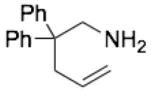


Yield: 2.5 g (10.5 mmol, 75%); colourless oil.

$^1$H NMR (300 MHz, $CDCl_3$): δ = 7.08-7.25 (m, 10H), 5.28-5.39 (m, 1H), 4.83-5.02 (m, 2H), 3.25 (m, 2H), 2.84-2.86 (d, $J$ = 6 Hz, 2H), 1.03 (s, 2H) ppm; Spectral data are in agreement with literature.[4]

**2,2-Dimethyl-pent-4-enylamine (S4)**

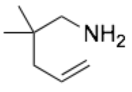


Yield: 0.9 g (7.9 mmol, 53%); yellow oil.

$^1$H NMR (400 MHz, $CDCl_3$): δ = 5.71-5.82 (m, 1 H), 4.95-7.99 (m, 2H), 2.38 (s, 2H), 2.03 (m, 2H), 0.87 (s, 6H) ppm; Spectral data are in agreement with literature.[5]

**Protection of amines**

The amine (6.5 mmol) was dissolved in 20 mL of dry $CH_2Cl_2$, and treated with tosyl chloride or benzoyl chloride or di-*tert*-butyl dicarbonate (7.1 mmol) and pyridine (1.5 mL, 19.4 mmol). The mixture was stirred at room temperature for 24 h. The solution was diluted with $H_2O$ (10 mL) and extracted with $Et_2O$ (3 x 10 mL). The combined organic layers were washed with brine (10 mL), dried over $MgSO_4$ and concentrated in vacuo. The crude material was purified by column chromatography on silica gel using ethyl acetate/hexane (1:9).

***N*-(2,2-Dimethyl-pent-4-enyl)-4-methyl-benzenesulfonamide (7)**

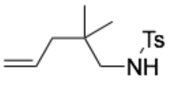


Yield: 0.266 g (0.99 mmol, 23%); colourless solid; m.p.: 73-74 ºC. $^1$H NMR (400 MHz, $CDCl_3$): δ = 7.72 (d, $J$ = 6 Hz, 2H), 7.30 (d, $J$ = 8 Hz, 2H), 5.70 (m, 1H), 5.02-4.90 (m, 2H), 4.98 (t, $J$ = 8 Hz, 1H), 2.68 (d, $J$ = 7 Hz, 2H), 2.40 (s, 3H), 1.99 (d, $J$ = 7 Hz, 2H), 0.85 (s, 6H) ppm; Spectral data are in agreement with literature.[6,7]

**4,4-Dimethyl-2-((phenylthio)methyl)-1-tosylpyrrolidine (8)**

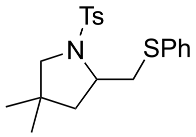


$^{1}$H NMR (400 MHz, $CDCl_3$): δ = 7.43 (d, *J* = 8.2 Hz, 2H), 7.28 (d, *J* = 8.2 Hz, 2H), 7.08-7.12 (m, 5H), 3.82 (dd, *J* = 13.3, 3.0 Hz, 1H), 3.55-3.48 (m, 1H), 3.12 (d, *J* = 10.5 Hz, 1H), 2.93 (d, *J* = 10.4 Hz, 1H), 2.78 (dd, *J* = 13.3, 10.4 Hz, 1H), 2.30 (s, 3H), 1.76 (dd, *J* = 12.8, 7.6 Hz, 1H), 1.53 (dd, *J* = 12.9, 7.8 Hz, 1H), 0.95 (s, 3H), 0.36 (s, 3H) ppm; $^{13}$C NMR (100 MHz, $CDCl_3$): δ = 142.4, 135.2, 132.9, 130.6, 129.1, 128.8, 128.5, 126.5, 60.9, 58.1, 44.9, 39.2, 36.2, 25.4, 24.8, 20.5 ppm.[8]

***N*-(2,2-Diphenyl-pent-4-enyl)-4-methyl-benzenesulfonamide (9)**

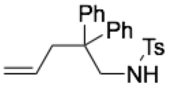


Yield: 2.0 g (5.1 mmol, 61%); colourless solid. $^{1}$H NMR (400 MHz, $CDCl_3$): δ = 7.61 (d, *J* = 9 Hz, 2H), 7.29-7.29 (m, 8H), 7.07 (d, *J* = 8 Hz, 4H), 5.21-5.32 (m, 1H), 4.92-4.96 (m, 2H), 3.82 (t, *J* = 8 Hz, 1H), 3.52 (d, *J* = 6 Hz, 2H), 2.90 (d, *J* = 7 Hz, 2H), 2.43 (s, 3H) ppm; Spectral data are in agreement with literature.[5]

**4,4-Diphenyl-2-((phenylthio)methyl)-1-tosylpyrrolidine (10)**

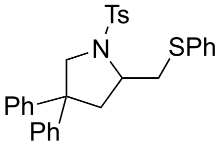


$^{1}$H NMR (400 MHz, $CDCl_3$): δ = 7.58-6.57 (m, 19H), 3.97 (d, *J* = 13.2 Hz, 1H), 3.27-3.07 (m, 3 H), 3.02 (dd, *J* = 12.3, 8.2 Hz, 1H), 2.64 (s, 1H), 2.50 (d, *J* = 12.3 Hz, 1H), 2.31 (s, 1H), 2.16-2.06 (m, 1H), 1.67 (ddd, *J* = 21.7, 10.9, 7.1 Hz, 2H) ppm; $^{13}$C NMR (100 MHz, $CDCl_3$): δ = 147.9, 146.9, 138.9, 136.1, 133.6, 130.05, 129.7, 129.6, 128.3, 128.2, 128.1, 127.9, 127.4, 127.2, 125.8, 125.7, 60.2, 59.4, 59.1, 46.4, 36.1, 33.3, 26.9 ppm; HRMS (ESI): calc. for $C_{30}H_{30}NO_2S_2$ $[M+H]^+$: 500.1640, found: 500.1648.

**4-Methyl-N-(pent-4-en-1-yl)benzenesulfonamide (11)**

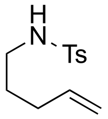

Prepared according to literature.[9]

**2-((Phenylthio)methyl)-1-tosylpyrrolidine (12)** [10]

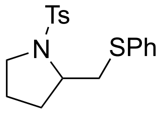

$^{1}$H NMR (400 MHz, $CDCl_3$): δ = 7.58 (d, J = 8.0 Hz, 2H), 7.49 (d, *J* = 7.5 Hz, 2H), 7.37 (t, *J* = 7.5 Hz, 2H), 7.28 – 7.21 (m, 3H), 3.71 (dd, *J* = 13, 3 Hz, 1H), 3.65 (ddt, *J* = 11, 7.2, 3.1 Hz, 1H), 3.51 (ddd, *J* = 10, 6.2, 4.2 Hz, 1H), 3.12 (ddd, *J* = 10.0, 8.0, 7 Hz, 1H), 2.79 (dd, *J* = 13.5, 11.0 Hz, 1H), 2.42 (s, 3H), 1.94-1.86 (m, 1H), 1.86-1.77 (m, 1H), 1.70-1.60 (m, 1H), 1.58-1.50 (m, 1H) ppm; $^{13}$C NMR (100 MHz, $CDCl_3$): δ = 143.4, 135.3, 133.7, 129.6, 129.0, 128.9, 127.4, 126.0, 58.8, 49.7, 38.3, 30.2, 23.7, 21.5 ppm.

**2-(((4-Chlorophenyl)thio)methyl)-1-tosylpyrrolidine (13)** [11]

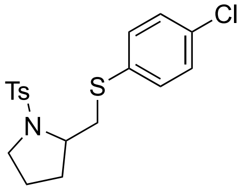

$^{1}$H NMR (400 MHz, $CDCl_3$): δ = 1.46-1.70 (m, 2H), 1.71-1.94 (m, 2H), 2.41 (s, 3H), 2.77 (dd, *J* = 13.4, 10.4 Hz, 1H), 3.03 (m, 1H), 3.57 (m, 2H), 3.64 (m, 1H), 7.26 (d, *J* = 8.0 Hz, 2H), 7.32 (d, *J* = 8.9 Hz, 2H), 7.40 (d, *J* = 8.9 Hz, 2H), 7.55 (d, *J* = 8.3 Hz, 2H) ppm; $^{13}$C NMR (100 MHz, $CDCl_3$): δ = 143.6, 133.9 , 133.7, 131.9, 130.2, 129.7, 129.1, 127.4, 58.7, 49.7, 38.5, 30.2, 23.7, 21.5 ppm.

**1-Methyl-2-(((1-tosylpyrrolidin-2-yl)methyl)thio)-1*H*-imidazole (14)** [12]

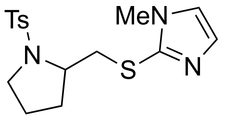

$^{1}$H NMR (400 MHz, [$D_6$]-DMSO): *δ* = 1.68-1.77 (m, 1H), 1.83-1.98 (m, 2H), 2.07-2.16 (m, 1H), 3.19-3.22 (m, 2H), 3.35–3.42 (m, 2 H), 3.61 (s, 3 H), 3.81–3.84 (m, 1 H), 7.01 (d, *J* = 1.6Hz, 1 H), 7.33 (d, *J* = 1.6 Hz, 1 H) ppm; $^{13}$C NMR (100 MHz, [$D_6$]-DMSO, 25 °C): δ = 23.4, 29.2, 33.2, 34.4, 44.6, 59.1, 123.6, 127.9, 139.7 ppm.

*Procedure for asymmetric cyclization:*

Into an oven-dried round-bottom flask under nitrogen a solution of *N*-(2-allylphenyl)-4-methylbenzenesulfamide **1a** (50 mg, 0.17 mmol) in dry $CH_2Cl_2$ (2 mL) was added dropwise to a suspension of **18a** (125 mg, 0.21 mmol) in dry $CH_2Cl_2$ (2 mL) at –20 °C. The reaction mixture was stirred for 15 min and then treated carefully with suspension of sodium benzenethiolate (25 mg, 0.19 mmol) in dry $CH_2Cl_2$ (2 mL). The reaction was stirred for further 15 min and quenched with saturated sodium thiosulfate solution (5 mL), diluted with water (5 mL) and extracted with $CH_2Cl_2$ (3 x 5 ml). The organic layers were combined, washed with brine (5 mL) and dried over $MgSO_4$, filtered and the solvent was carefully removed under reduced pressure. The crude material was purified by column chromatography on silica gel using ethyl acetate/hexane (20%).

**(*R*)-2-((Phenylthio)methyl)-1-tosylindoline [(*R*)-2a]**

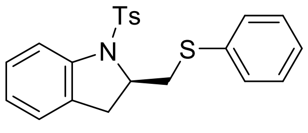


$[\alpha]^{20}_{D}$ = 11.5 (c = 0.5, $CHCl_3$)

HPLC analysis: Daicel Chiralcel AD-H column, hexane/*i*-PrOH = 94/6, 0.8 mL/min, 254 nm; $t_R$ (*S*) = 18.78 min, $t_R$ (*R*) = 21.21 min; 79% *ee*.

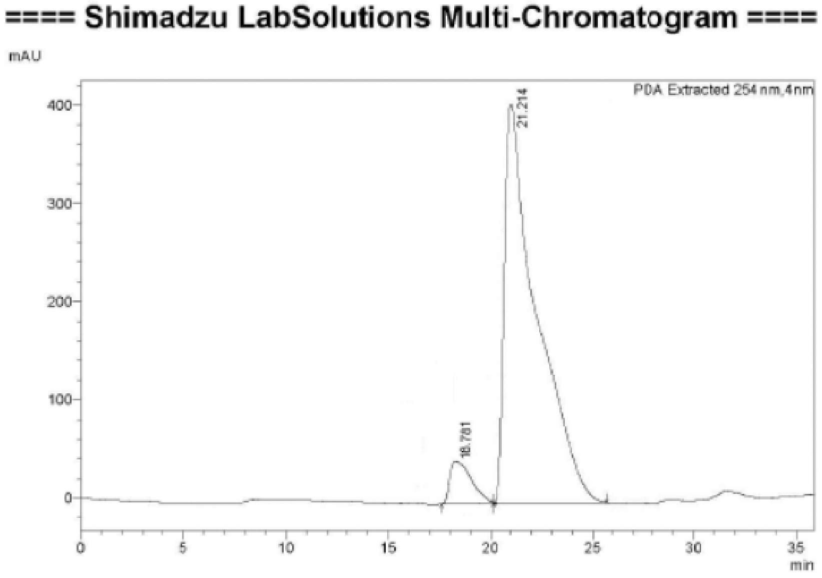


| Peak # | Ret.Time | Area % |
|---|---|---|
| 1 | 18.781 | 10.59 |
| 2 | 21.214 | 89.41 |
| | | 78.82 |

**(*R*)-5-Chloro-2-((phenylthio)methyl)-1-tosylindoline [(*R*)-4]**

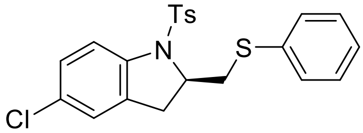


$[\alpha]^{20}_{D}$ = 12.2 (c = 0.5, $CHCl_3$)

HPLC analysis: Daicel Chiralcel AD-H column, hexane/*i*-PrOH = 95/5, 0.8 mL/min, 275nm; $t_R$ (*S*) = 19.91 min), $t_R$ (*R*) = 22.43 min; 74% *ee* [absolute stereochemistry assigned in comparison to the literature: (*S*)-5-fluoro-2-((phenylthio)methyl)-1-tosylindoline $[\alpha]^{25}_{D}$ = –15.5 (c = 0.57, $CHCl_3$)].[1]

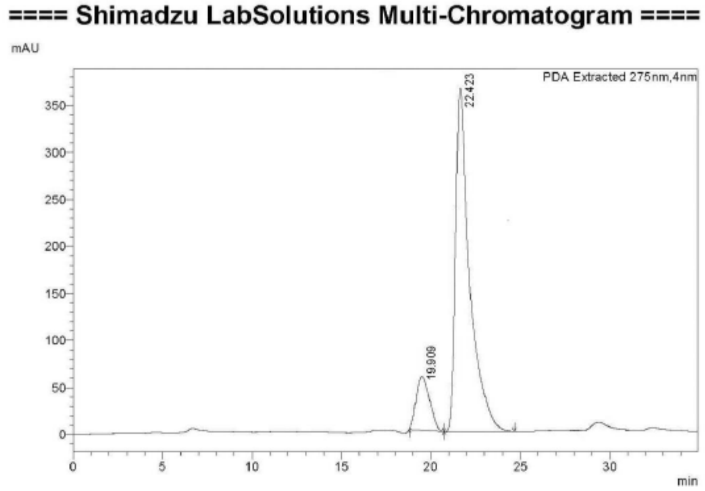


| Peak # | Ret.Time | Area % |
|---|---|---|
| 1 | 19.909 | 13.02 |
| 2 | 22.423 | 86.98 |
| | | 73.96 |

**(*R*)-5-Methoxy-2-((phenylthio)methyl)-1-tosylindoline [(*R*)-6]**

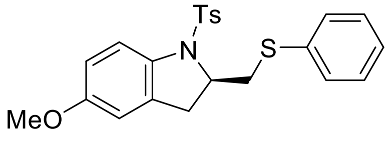


$[\alpha]^{20}_{D}$ = 9.3 (c = 0.5, $CHCl_3$)

HPLC analysis: Daicel Chiralcel AD-H column, hexane/*i*-PrOH = 98/2, 1.5 mL/min, 260 nm; $t_R$ (*S*) = 6.02 min), $t_R$ (*R*) = 8.14 min; 70% *ee* [absolute stereochemistry assigned in comparison to the literature: (*S*)-5-fluoro-2-((phenylthio)methyl)-1-tosylindoline $[\alpha]^{25}_{D}$ = –15.5 (c = 0.57, $CHCl_3$)].[1]

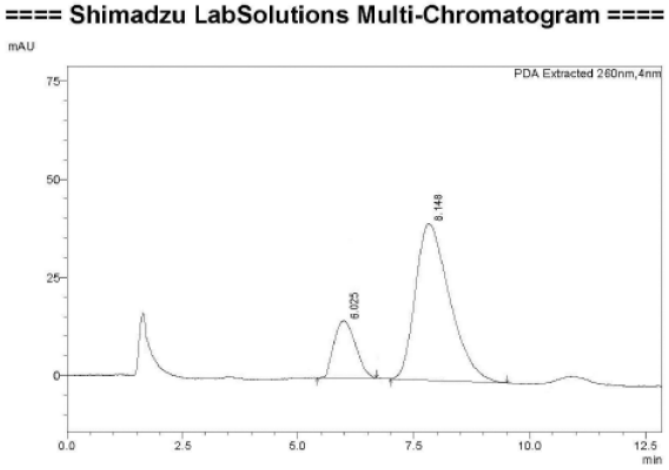


| Peak # | Ret.Time | Area % |
|---|---|---|
| 1 | 6.025 | 15.21 |
| 2 | 8.148 | 84.79 |
| | | 69.58 |

**(*R*)-4,4-Dimethyl-2-((phenylthio)methyl)-1-tosylpyrrolidine [(*R*)-8]**

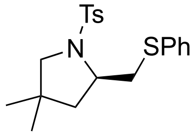


$[\alpha]^{20}_{D}$ = 98.7 (c = 0.5, $CHCl_3$)

HPLC analysis: Daicel Chiralcel OD column, hexane/*i*-PrOH = 90/10, 1 mL/min, 220 nm; $t_R$ (*S*) = 16.32 min, $t_R$ (*R*) = 18.52 min; 60% *ee* (absolute stereochemistry assigned in analogy to **(*R*)-12**).

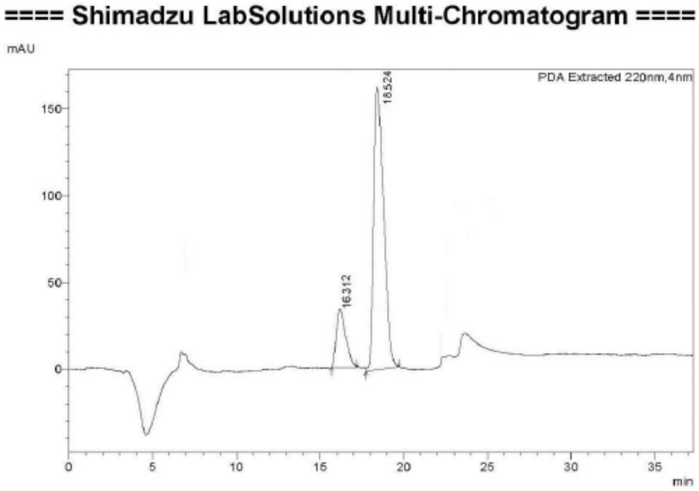


| Peak # | Ret.Time | Area % |
|---|---|---|
| 1 | 16.312 | 19.97 |
| 2 | 18.524 | 80.03 |
| | | 60.06 |

**(*R*)-4,4-Diphenyl-2-((phenylthio)methyl)-1-tosylpyrrolidine [(*R*)-10]**

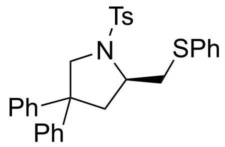


$[\alpha]^{20}_{D}$ = 69.8 (c = 0.5, $CHCl_3$)

HPLC analysis: Daicel Chiralcel OD column, hexane/*i*-PrOH = 92/8, 1 mL/min, 220 nm; $t_R$ (*S*) = 14.58 min, $t_R$ (*R*) = 17.05 min; 61% *ee* (absolute stereochemistry assigned in analogy to **(*R*)-12**).

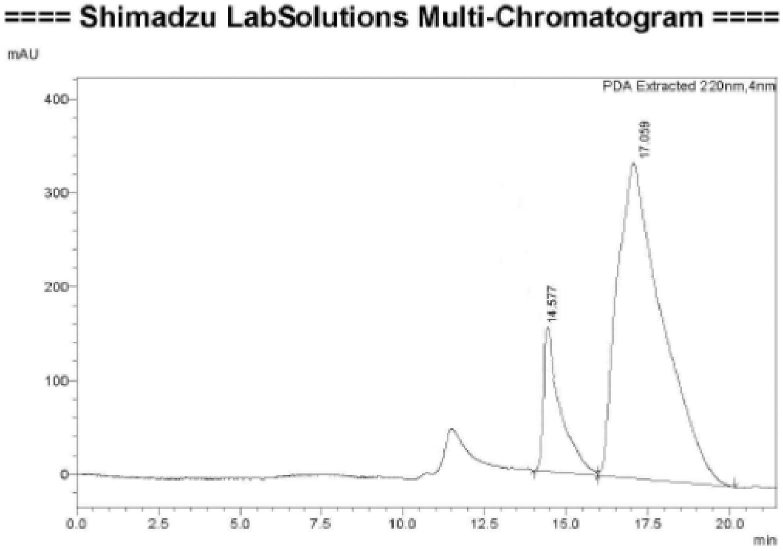


| Peak # | Ret.Time | Area % |
|---|---|---|
| 1 | 14.577 | 19.31 |
| 2 | 17.059 | 80.69 |
| | | 61.38 |

**(*R*)-2-((Phenylthio)methyl)-1-tosylpyrrolidine [(*R*)-12]**

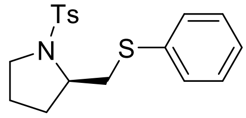


$[\alpha]^{20}_{D}$ = 127.3 (c = 1, $CHCl_3$)

[Literature value: (*S*)-((phenylthio)methyl)-1-tosylpyrrolidine (85% *ee*): $[\alpha]^{24}_{D}$ = –228.7° (c = 1, $CHCl_3$)][10]

HPLC analysis: Daicel Chiralcel OD-H column, hexane/*i*-PrOH = 95/5, 0.8 mL/min, 220 nm; $t_R$ (*S*) = 13.11 min, $t_R$ (*R*) = 15.21 min; 55% *ee*.

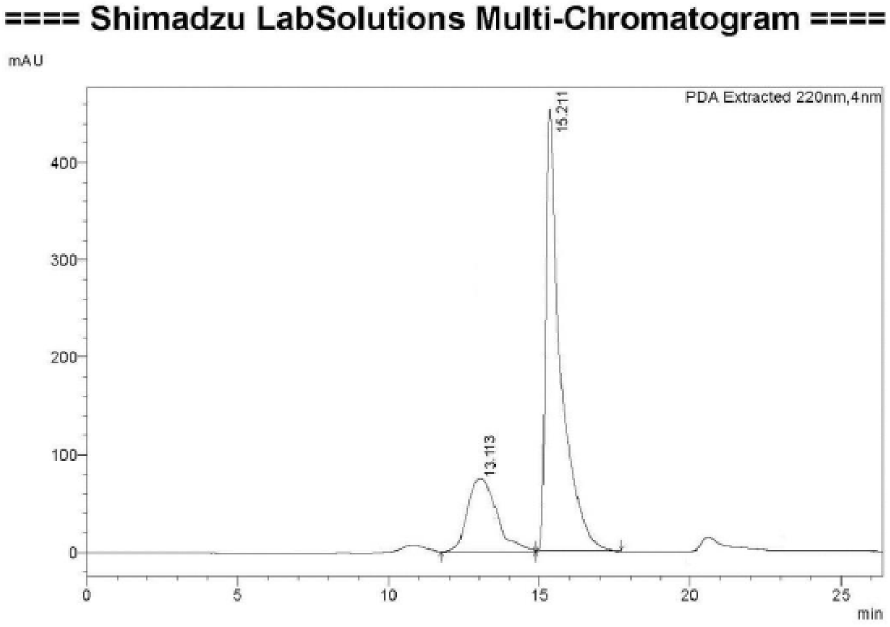


| Peak # | Ret.Time | Area % |
|---|---|---|
| 1 | 13.113 | 22.69 |
| 2 | 15.211 | 77.31 |
| | | 54.62 |

**(*R*)-1-Methyl-2-(((1-tosylpyrrolidin-2-yl)methyl)thio)-1*H*-imidazole [(*R*)-14]**

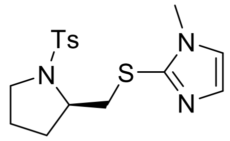


$[\alpha]^{20}_{D}$ = 32.1 (c = 1, $CHCl_3$)

HPLC analysis: Daicel Chiralpak OD-H, hexane/*i*PrOH = 95:5, flow rate 1.0 mL/min, 254 nm: $t_R$ (*S*) = 15.81, $t_R$ (*R*) 20.05 min; 25% *ee* (absolute stereochemistry assigned in analogy to **(*R*)-12**).

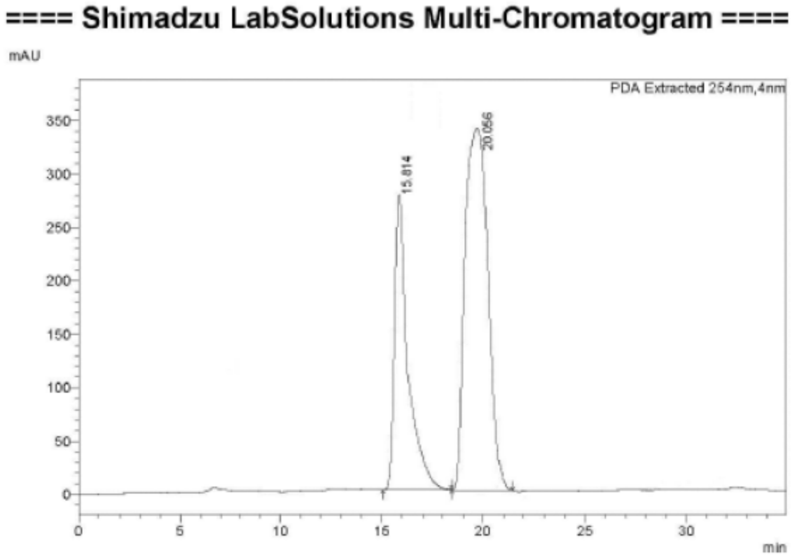


| Peak # | Ret.Time | Area % |
|---|---|---|
| 1 | 15.814 | 37.57 |
| 2 | 20.056 | 62.43 |
| | | 24.86 |


[1] M. T. Bovino, S. R. Chemler, *Angew. Chem. Int. Ed.* **2012**, *51*, 3923-3927.

[2] F. E. Michael, B. M. Cochran, *J. Am. Chem. Soc.* **2006**, *128*, 4246-4247.

[3] X. Han, R. A. Widenhoefer, *Angew. Chem. Int. Ed.* **2006**, *45*, 1747-1749.

[4] P. H. Martínez, K. C. Hultzsch, F. Hampel, *Chem. Commun.* **2006**, 2221-2223.

[5] P. H. Fuller, J. W. Kim, S. R. Chemler, *J. Am. Chem. Soc.* **2009**, *130*, 17638-17639.

[6] C. F. Bender, R. A. Widenhoefer, *J. Am. Chem. Soc.* **2005**, *127*, 1070-1071.

[7] W. Zeng, S. R. Chemler, *J. Am. Chem. Soc.* **2007**, *129*, 12948-12949.

[8] G. Liu, Y. Li, *J. Org. Chem.* **2014**, *79*, 10094-10109.

[9] G.-Q. Liu, Z.-Y. Ding, L. Zhang, T.-T. Li, L. Li, L. Duan, Y.-M. Li, *Adv. Synth. Catal.* **2014**, *356*, 2303-2310.

[10] S. E. Denmark, H. M. Chi, *J. Am. Chem. Soc.* **2014**, *136*, 8915-8918.

[11] V. Hugenberg, R. Fröhlich, G. Haufe, *Org. Biomol. Chem.,* **2010**, *8*, 5682-5691.

[12] D. Xu, L.Wang, S. Luo, Y.Wang, S. Zhang, Z. Xu, *Eur. J. Org. Chem.* **2008**, 1049-1053.

NMR spectra

**2-((Phenylthio)methyl)-1-tosylindoline (2a)**

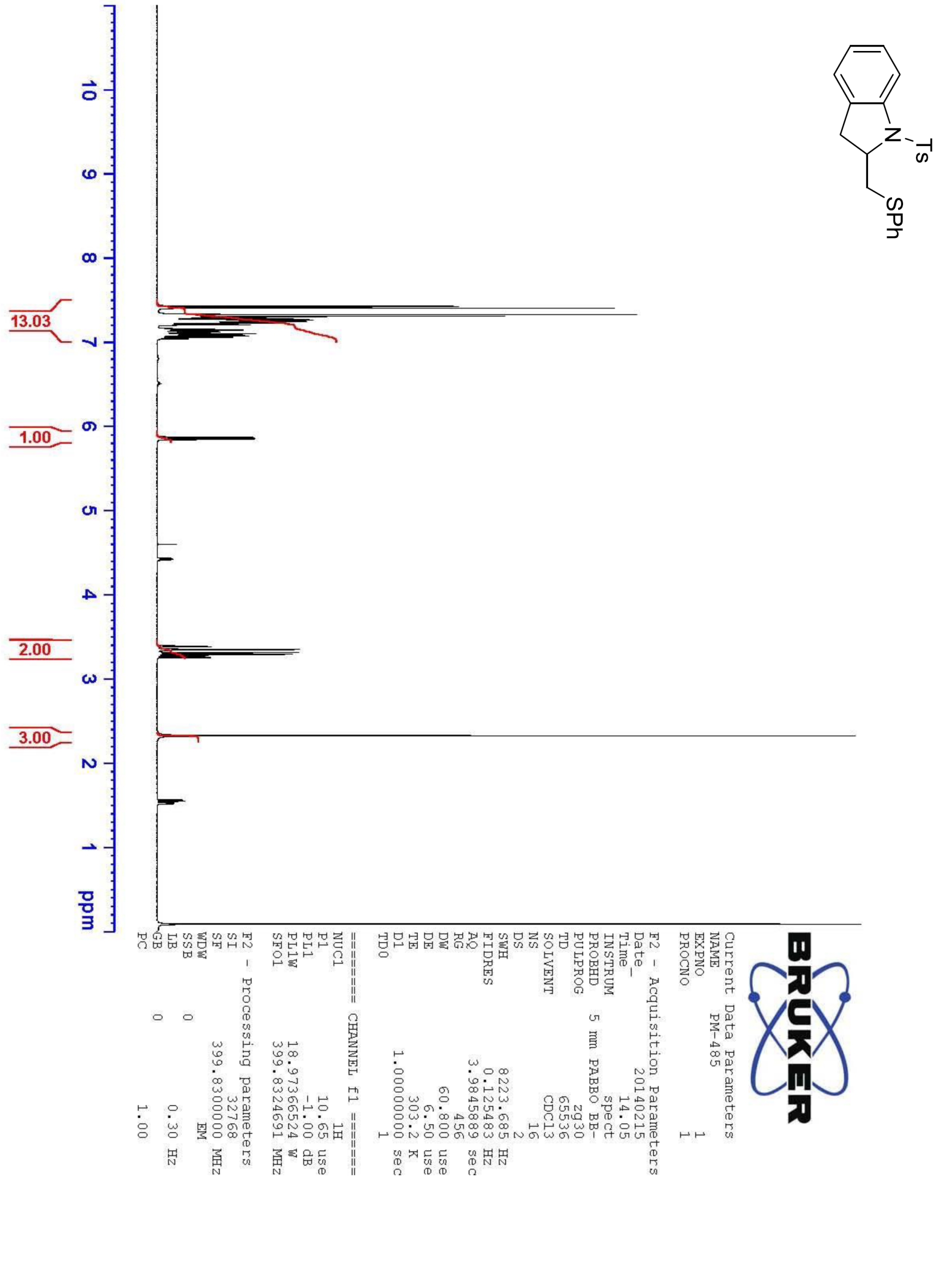

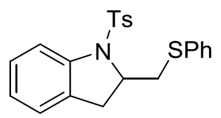
Ts
N
SPh

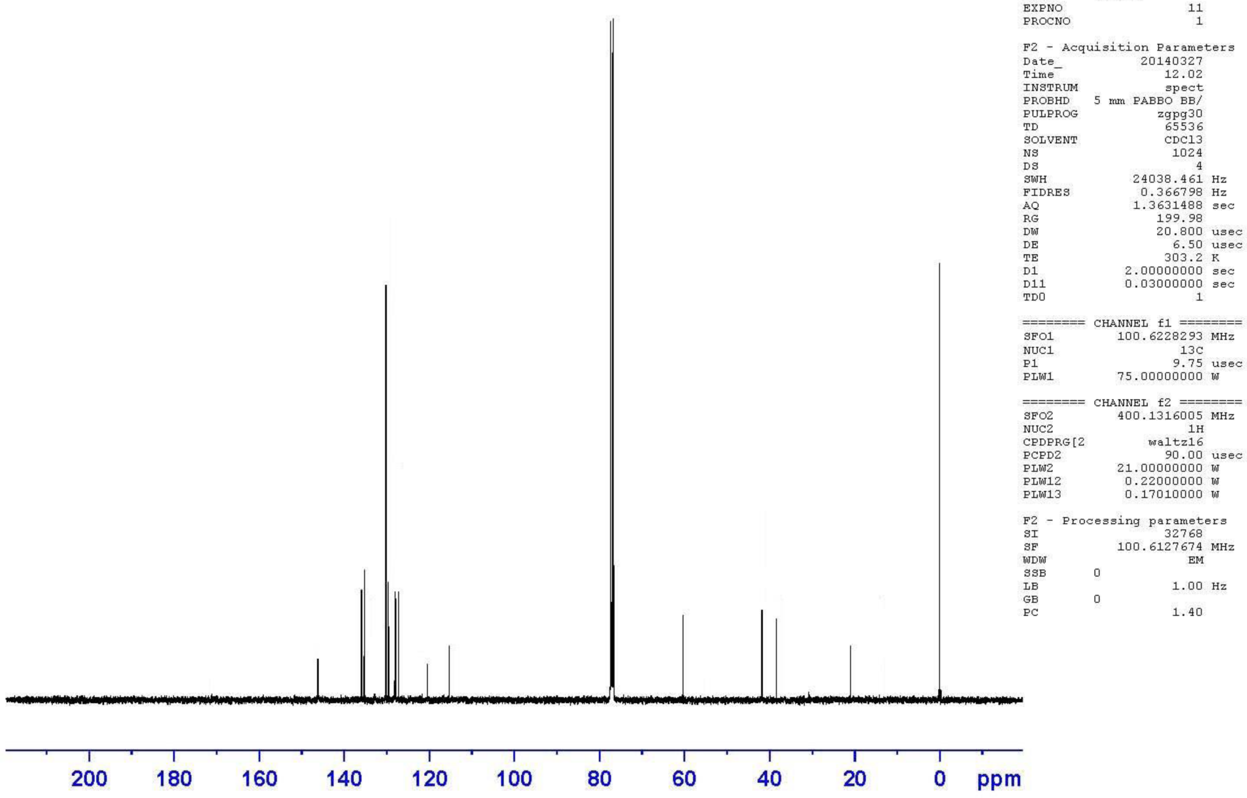
0.25
-0.00
-0.26
BRUKER
Current Data Parameters
NAME PM-662
EXPNO 11
PROCNO 1
F2 - Acquisition Parameters
Date_ 20140327
Time 12.02
INSTRUM spect
PROBHD 5 mm PABBO BB/
PULPROG zgpg30
TD 65536
SOLVENT CDCl3
NS 1024
DS 4
SWH 24038.461 Hz
FIDRES 0.366798 Hz
AQ 1.3631488 sec
RG 199.98
DW 20.800 usec
DE 6.50 usec
TE 303.2 K
D1 2.00000000 sec
D11 0.03000000 sec
TD0 1
======== CHANNEL f1 ========
SFO1 100.6228293 MHz
NUC1 13C
P1 9.75 usec
PLW1 75.00000000 W
======== CHANNEL f2 ========
SFO2 400.1316005 MHz
NUC2 1H
CPDPRG[2 waltz16
PCPD2 90.00 usec
PLW2 21.00000000 W
PLW12 0.22000000 W
PLW13 0.17010000 W
F2 - Processing parameters
SI 32768
SF 100.6127674 MHz
WDW EM
SSB 0
LB 1.00 Hz
GB 0
PC 1.40
200
180
160
140
120
100
80
60
40
20
0
ppm

## 5-Chloro-2-((phenylthio)methyl)-1-tosylindoline (4)

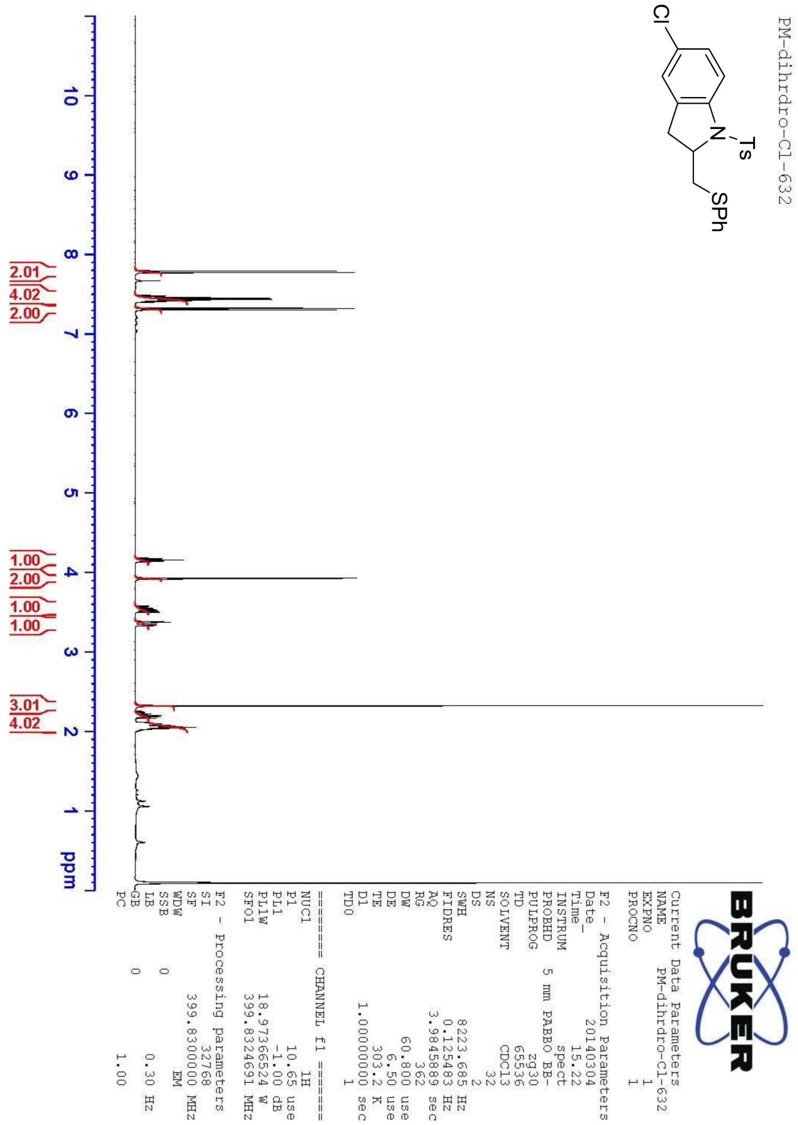

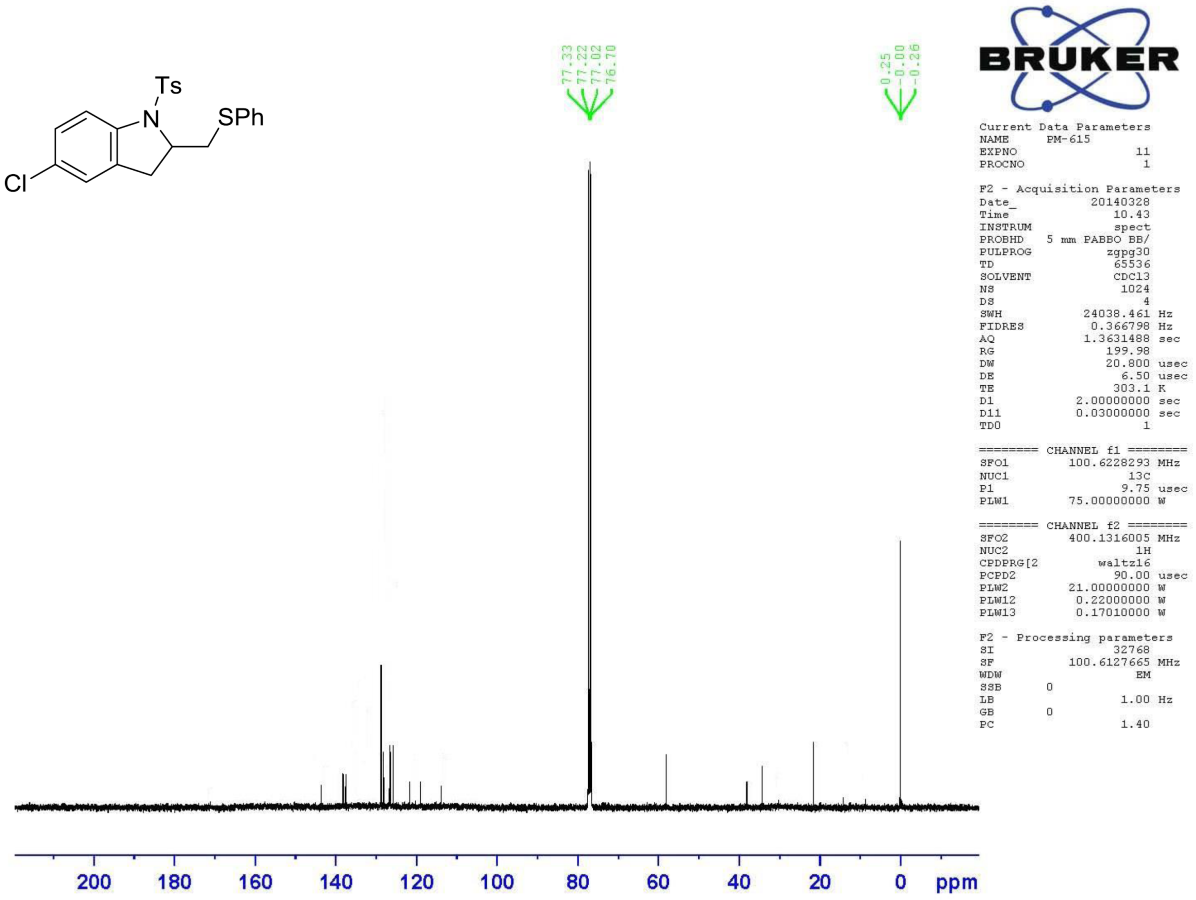
Ts
N
SPh
Cl
77.33
77.22
77.02
76.70
0.25
-0.00
-0.26
BRUKER
Current Data Parameters
NAME PM-615
EXPNO 11
PROCNO 1
F2 - Acquisition Parameters
Date_ 20140328
Time 10.43
INSTRUM spect
PROBHD 5 mm PABBO BB/
PULPROG zgpg30
TD 65536
SOLVENT CDCl3
NS 1024
DS 4
SWH 24038.461 Hz
FIDRES 0.366798 Hz
AQ 1.3631488 sec
RG 199.98
DW 20.800 usec
DE 6.50 usec
TE 303.1 K
D1 2.00000000 sec
D11 0.03000000 sec
TD0 1
======== CHANNEL f1 ========
SFO1 100.6228293 MHz
NUC1 13C
P1 9.75 usec
PLW1 75.00000000 W
======== CHANNEL f2 ========
SFO2 400.1316005 MHz
NUC2 1H
CPDPRG[2 waltz16
PCPD2 90.00 usec
PLW2 21.00000000 W
PLW12 0.22000000 W
PLW13 0.17010000 W
F2 - Processing parameters
SI 32768
SF 100.6127665 MHz
WDW EM
SSB 0
LB 1.00 Hz
GB 0
PC 1.40
200
180
160
140
120
100
80
60
40
20
0
ppm

**5-Methoxy-2-((phenylthio)methyl)-1-tosylindoline (6)**

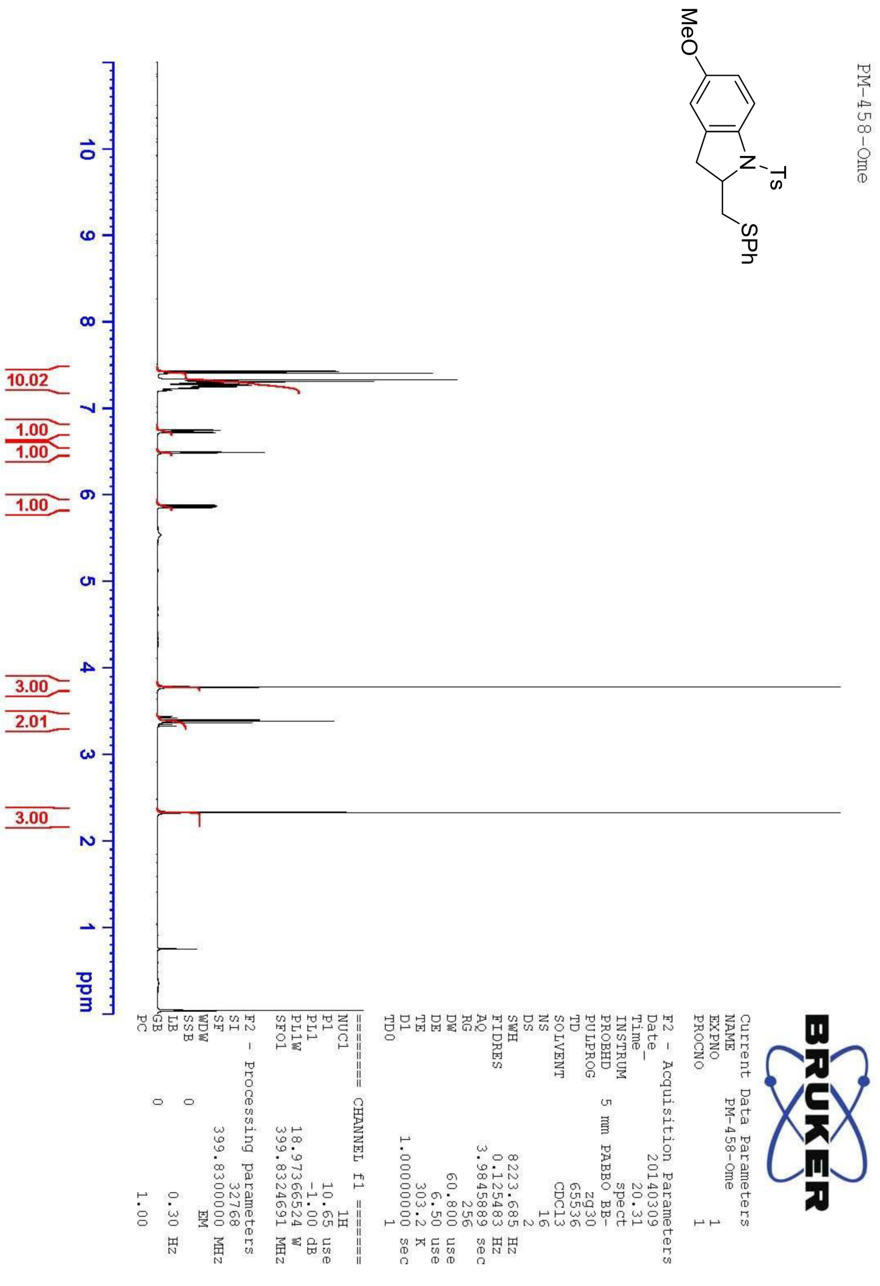

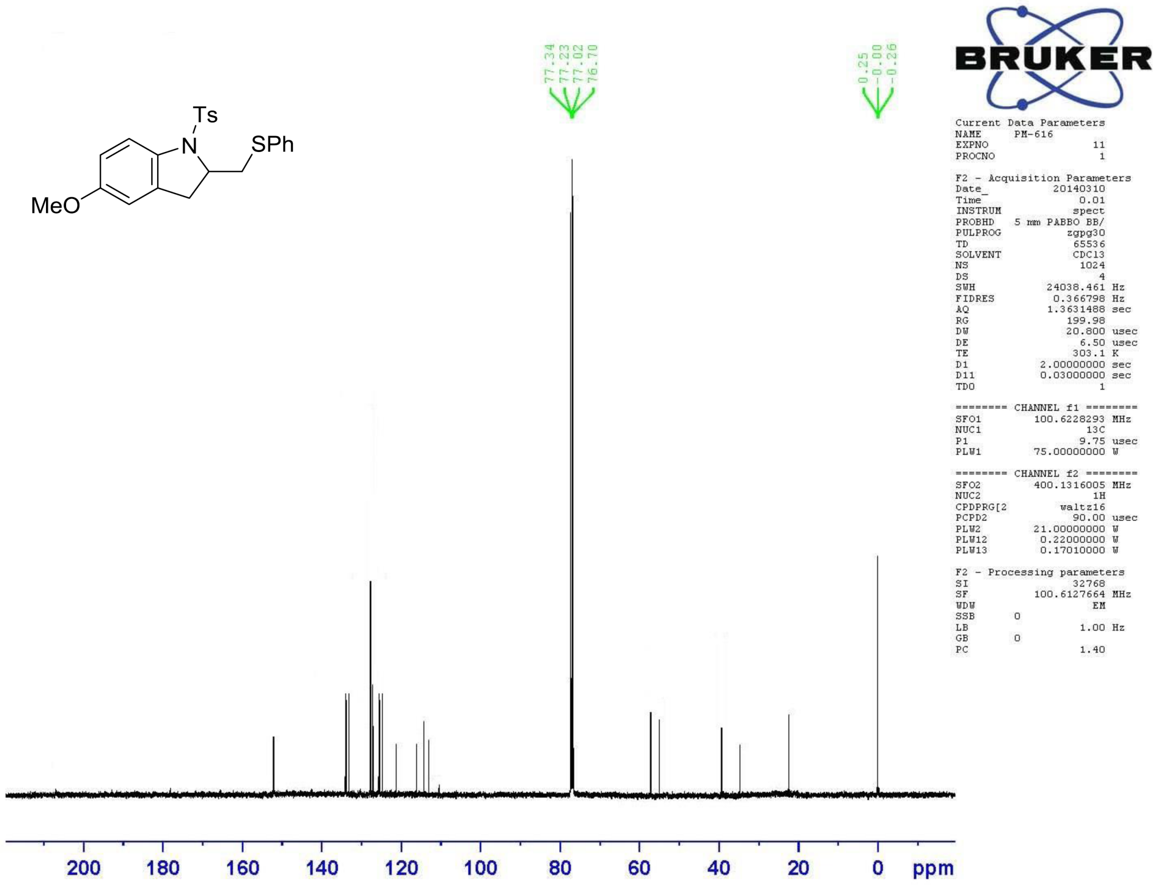

Ts
N
SPh
MeO
77.34
77.23
77.02
76.70
0.25
-0.00
-0.26
BRUKER
Current Data Parameters
NAME PM-616
EXPNO 11
PROCNO 1
F2 - Acquisition Parameters
Date_ 20140310
Time 0.01
INSTRUM spect
PROBHD 5 mm PABBO BB/
PULPROG zgpg30
TD 65536
SOLVENT CDCl3
NS 1024
DS 4
SWH 24038.461 Hz
FIDRES 0.366798 Hz
AQ 1.3631488 sec
RG 199.98
DW 20.800 usec
DE 6.50 usec
TE 303.1 K
D1 2.00000000 sec
D11 0.03000000 sec
TD0 1
======== CHANNEL f1 ========
SFO1 100.6228293 MHz
NUC1 13C
P1 9.75 usec
PLW1 75.00000000 W
======== CHANNEL f2 ========
SFO2 400.1316005 MHz
NUC2 1H
CPDPRG[2 waltz16
PCPD2 90.00 usec
PLW2 21.00000000 W
PLW12 0.22000000 W
PLW13 0.17010000 W
F2 - Processing parameters
SI 32768
SF 100.6127664 MHz
WDW EM
SSB 0
LB 1.00 Hz
GB 0
PC 1.40
200
180
160
140
120
100
80
60
40
20
0
ppm

## 4,4-Dimethyl-2-((phenylthio)methyl)-1-tosylpyrrolidine (8)

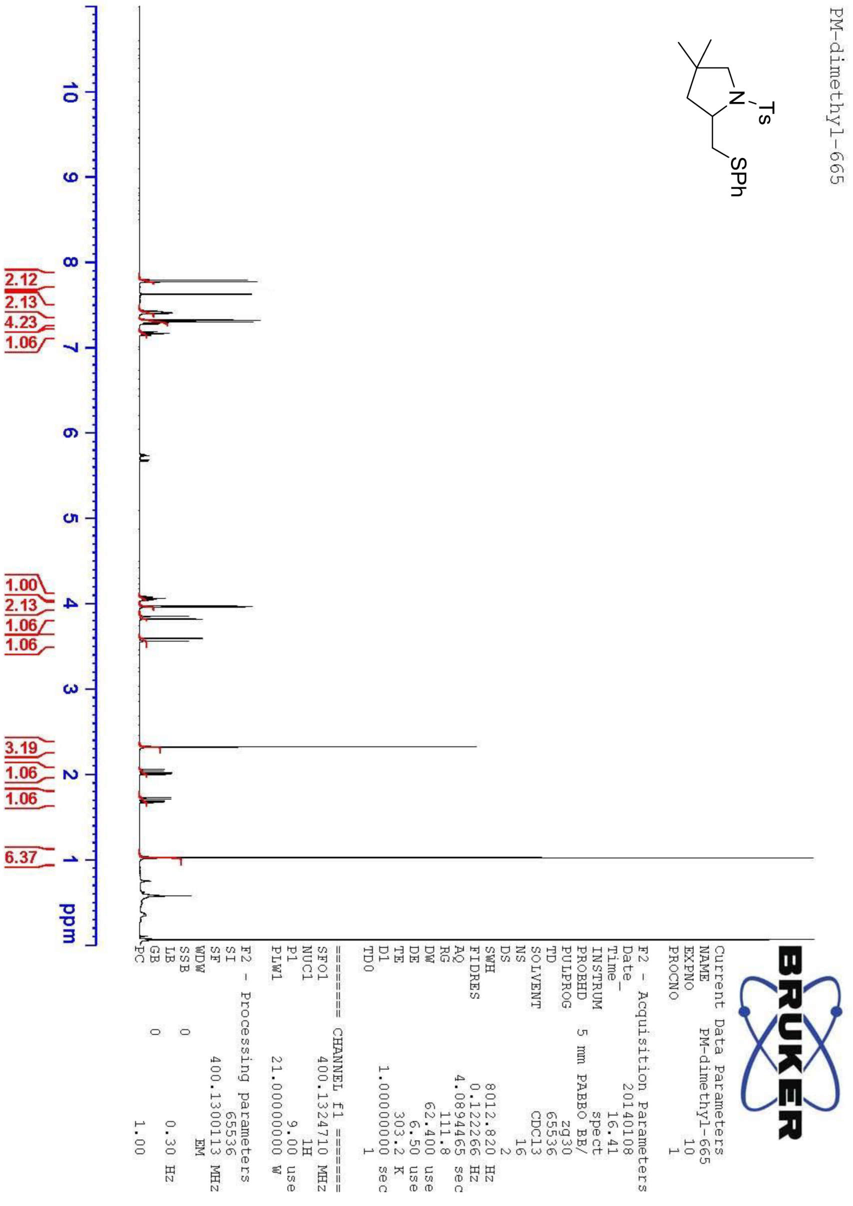

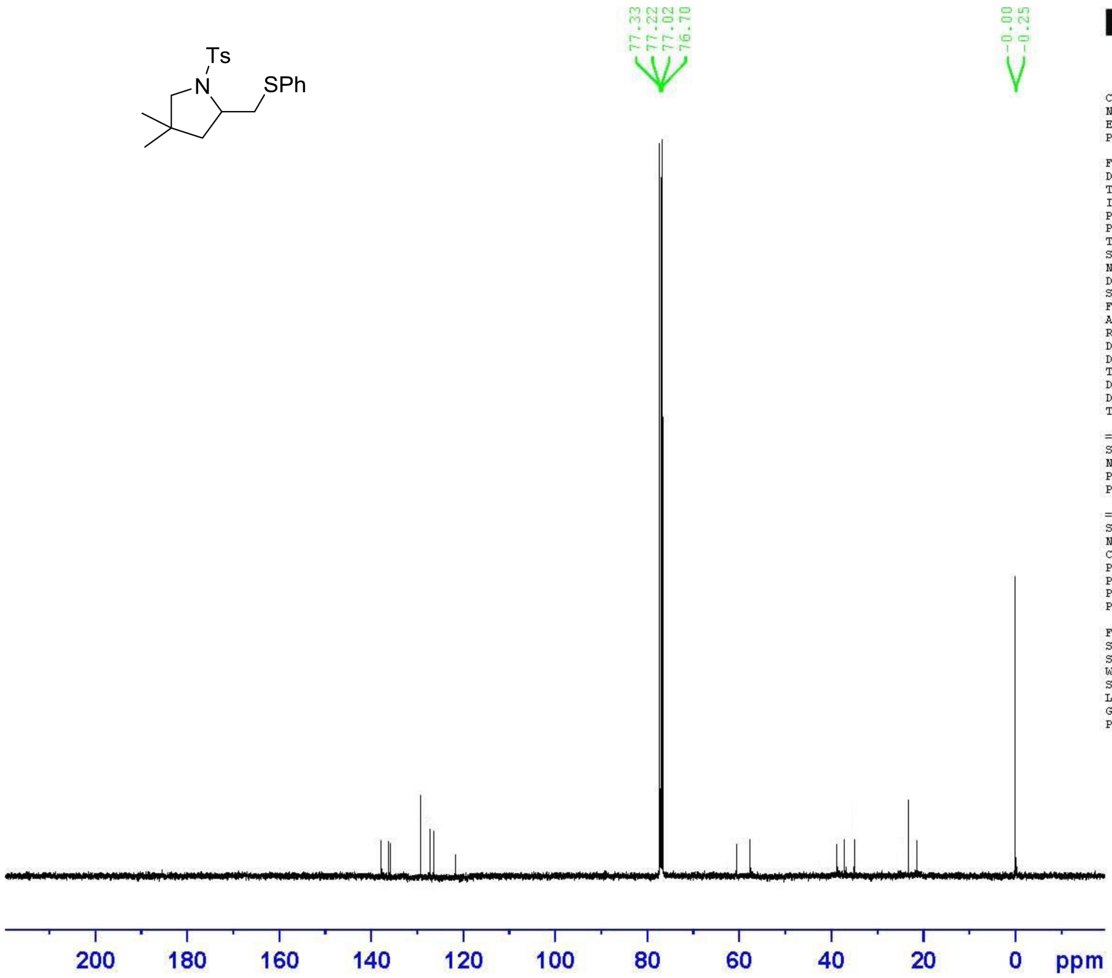


```
Current Data Parameters
NAME      PM-696
EXPNO               11
PROCNO               1

F2 - Acquisition Parameters
Date_         20140319
Time             20.54
INSTRUM          spect
PROBHD   5 mm PABBO BB/
PULPROG        zgpg30
TD              65536
SOLVENT         CDCl3
NS               1024
DS                  4
SWH         24038.461 Hz
FIDRES       0.366798 Hz
AQ          1.3631488 sec
RG             199.98
DW             20.800 usec
DE               6.50 usec
TE              303.2 K
D1         2.00000000 sec
D11        0.03000000 sec
TD0                 1

======== CHANNEL f1 ========
SFO1      100.6228293 MHz
NUC1              13C
P1               9.75 usec
PLW1      75.00000000 W

======== CHANNEL f2 ========
SFO2      400.1316005 MHz
NUC2               1H
CPDPRG[2       waltz16
PCPD2           90.00 usec
PLW2      21.00000000 W
PLW12      0.22000000 W
PLW13      0.17010000 W

F2 - Processing parameters
SI              32768
SF        100.6127667 MHz
WDW                EM
SSB        0
LB               1.00 Hz
GB         0
PC               1.40
```

**4,4-Diphenyl-2-((phenylthio)methyl)-1-tosylpyrrolidine (10)**

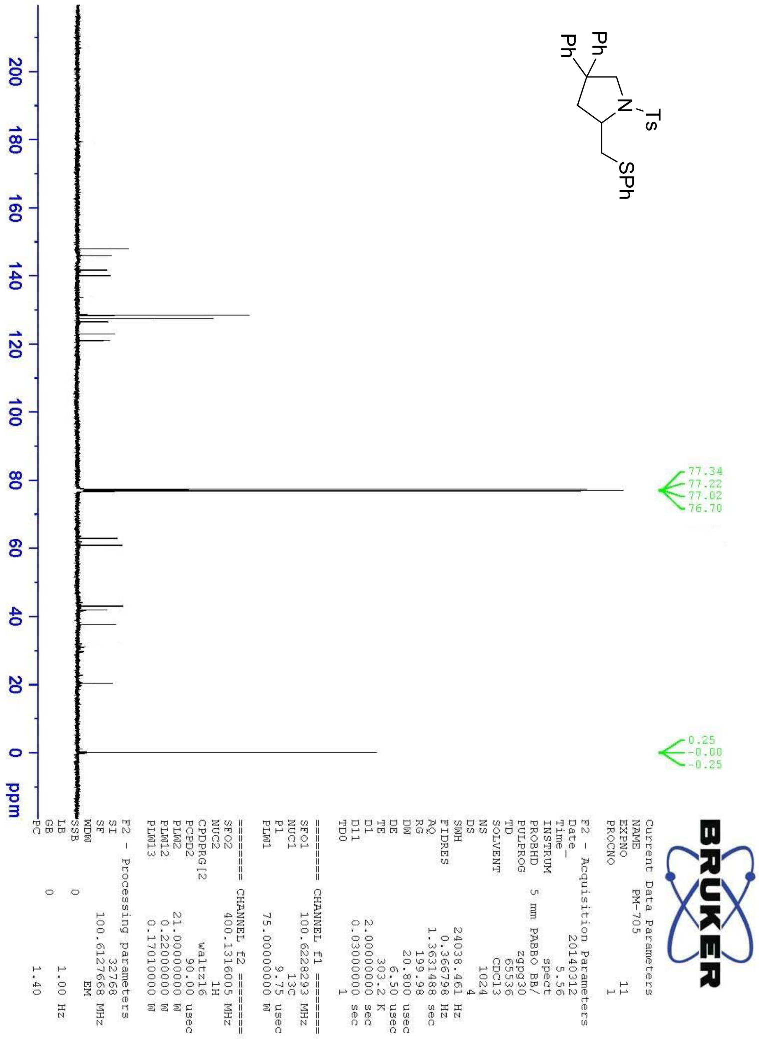

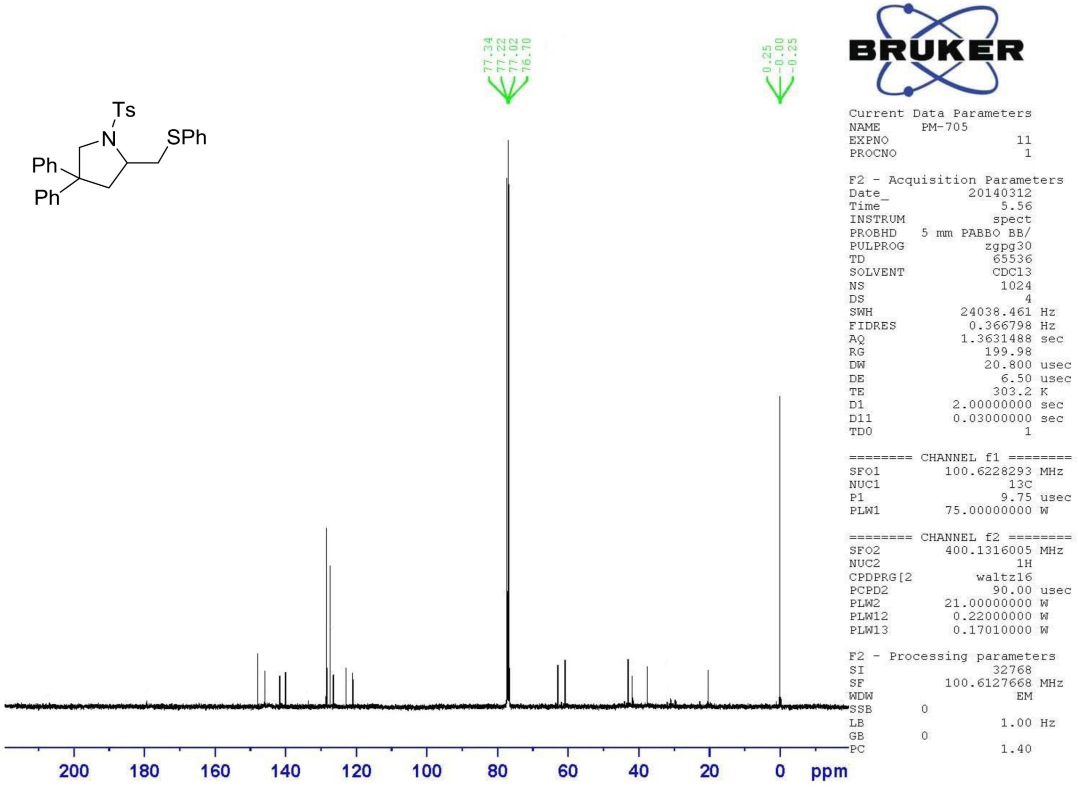
Ts
N
SPh
Ph
Ph
77.34
77.22
77.02
76.70
0.25
-0.00
-0.25
BRUKER
Current Data Parameters
NAME PM-705
EXPNO 11
PROCNO 1
F2 - Acquisition Parameters
Date_ 20140312
Time_ 5.56
INSTRUM spect
PROBHD 5 mm PABBO BB/
PULPROG zgpg30
TD 65536
SOLVENT CDCl3
NS 1024
DS 4
SWH 24038.461 Hz
FIDRES 0.366798 Hz
AQ 1.3631488 sec
RG 199.98
DW 20.800 usec
DE 6.50 usec
TE 303.2 K
D1 2.00000000 sec
D11 0.03000000 sec
TD0 1
======== CHANNEL f1 ========
SFO1 100.6228293 MHz
NUC1 13C
P1 9.75 usec
PLW1 75.00000000 W
======== CHANNEL f2 ========
SFO2 400.1316005 MHz
NUC2 1H
CPDPRG[2 waltz16
PCPD2 90.00 usec
PLW2 21.00000000 W
PLW12 0.22000000 W
PLW13 0.17010000 W
F2 - Processing parameters
SI 32768
SF 100.6127668 MHz
WDW EM
SSB 0
LB 1.00 Hz
GB 0
PC 1.40
200
180
160
140
120
100
80
60
40
20
0
ppm

**2-((Phenylthio)methyl)-1-tosylpyrrolidine (12)**

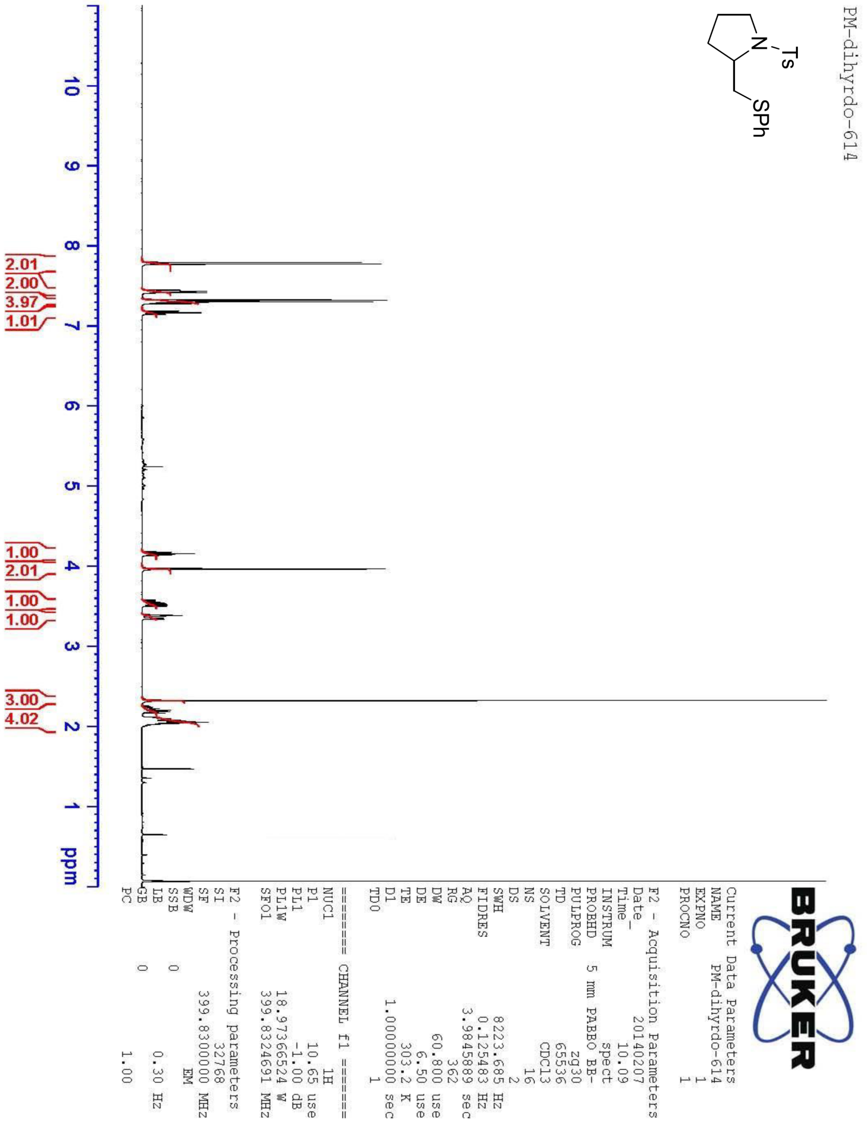

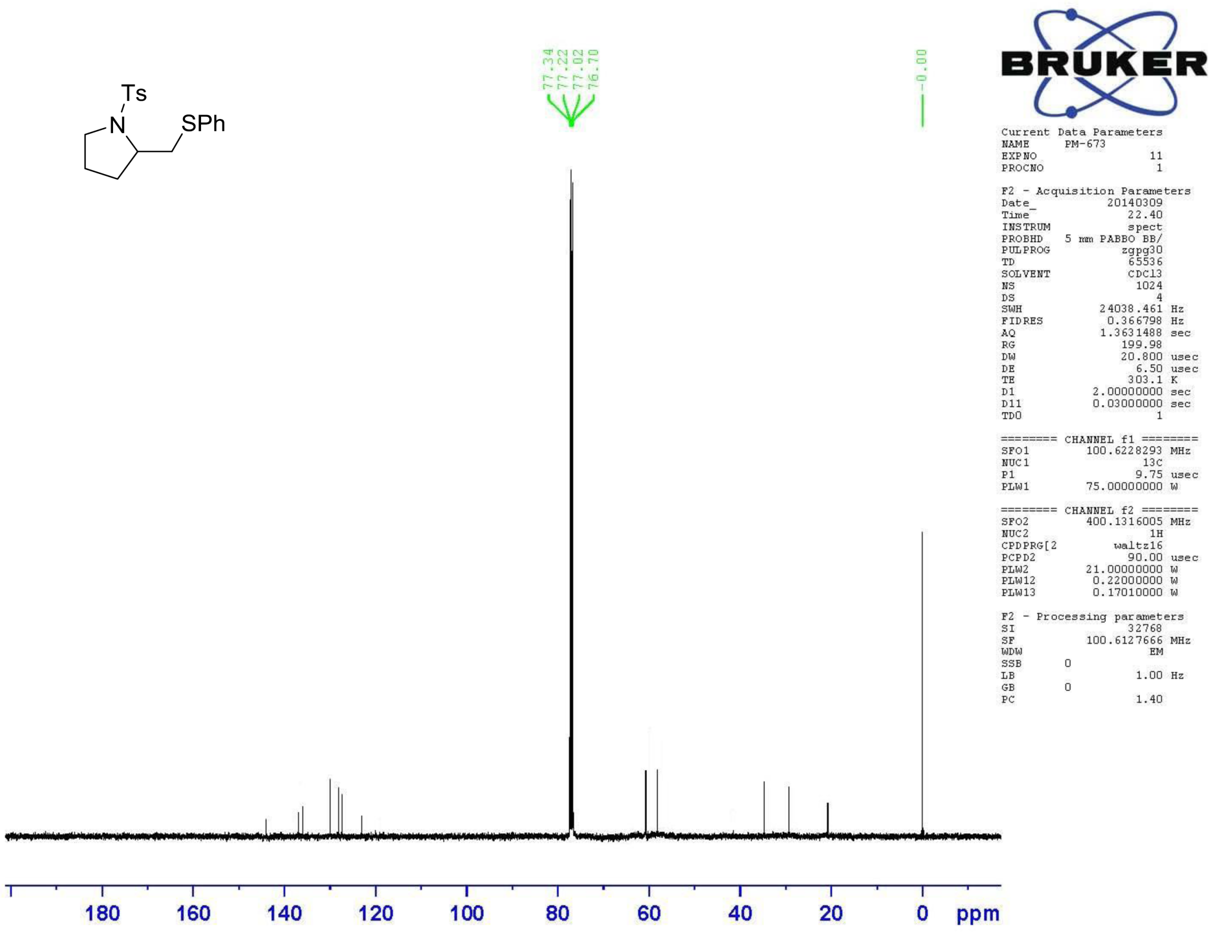
Ts
N
SPh
77.34
77.22
77.02
76.70
-0.00
BRUKER
Current Data Parameters
NAME PM-673
EXPNO 11
PROCNO 1
F2 - Acquisition Parameters
Date_ 20140309
Time 22.40
INSTRUM spect
PROBHD 5 mm PABBO BB/
PULPROG zgpg30
TD 65536
SOLVENT CDCl3
NS 1024
DS 4
SWH 24038.461 Hz
FIDRES 0.366798 Hz
AQ 1.3631488 sec
RG 199.98
DW 20.800 usec
DE 6.50 usec
TE 303.1 K
D1 2.00000000 sec
D11 0.03000000 sec
TD0 1
======== CHANNEL f1 ========
SFO1 100.6228293 MHz
NUC1 13C
P1 9.75 usec
PLW1 75.00000000 W
======== CHANNEL f2 ========
SFO2 400.1316005 MHz
NUC2 1H
CPDPRG[2 waltz16
PCPD2 90.00 usec
PLW2 21.00000000 W
PLW12 0.22000000 W
PLW13 0.17010000 W
F2 - Processing parameters
SI 32768
SF 100.6127666 MHz
WDW EM
SSB 0
LB 1.00 Hz
GB 0
PC 1.40
180
160
140
120
100
80
60
40
20
0
ppm

**1-Methyl-2-(((1-tosylpyrrolidin-2-yl)methyl)thio)-1*H*-imidazole (14)**

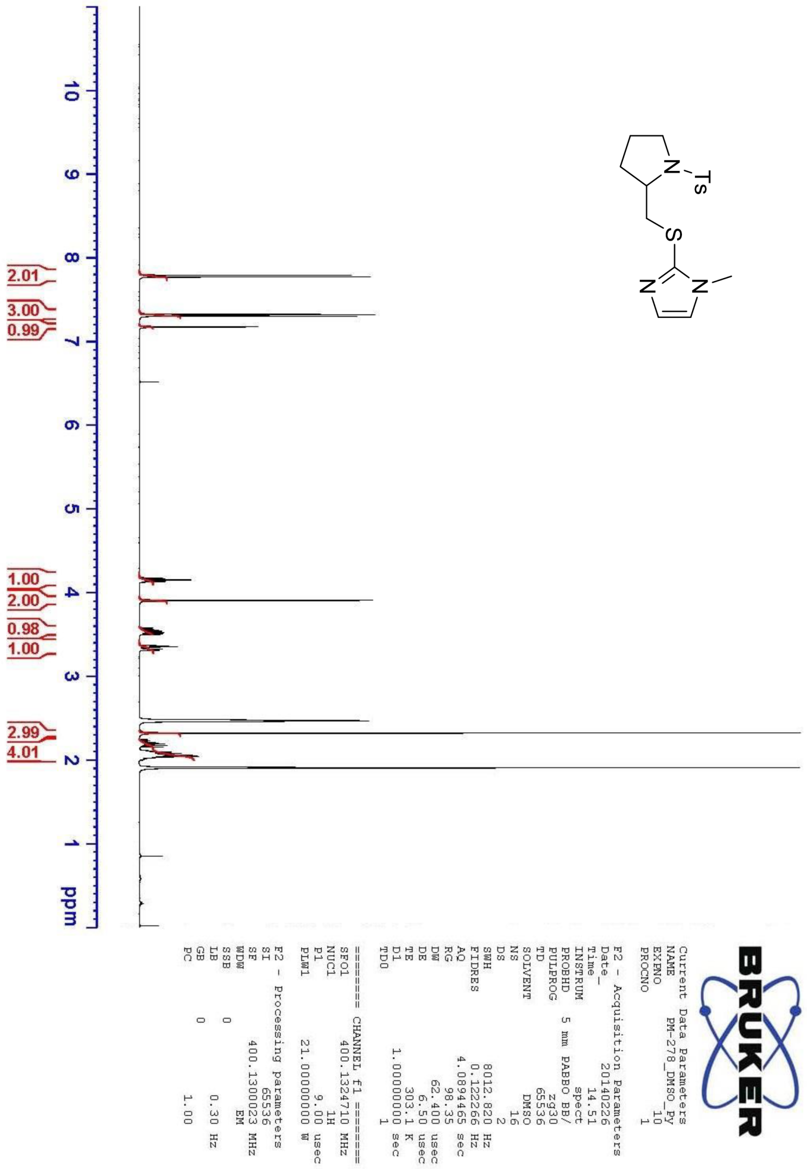

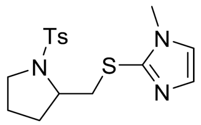


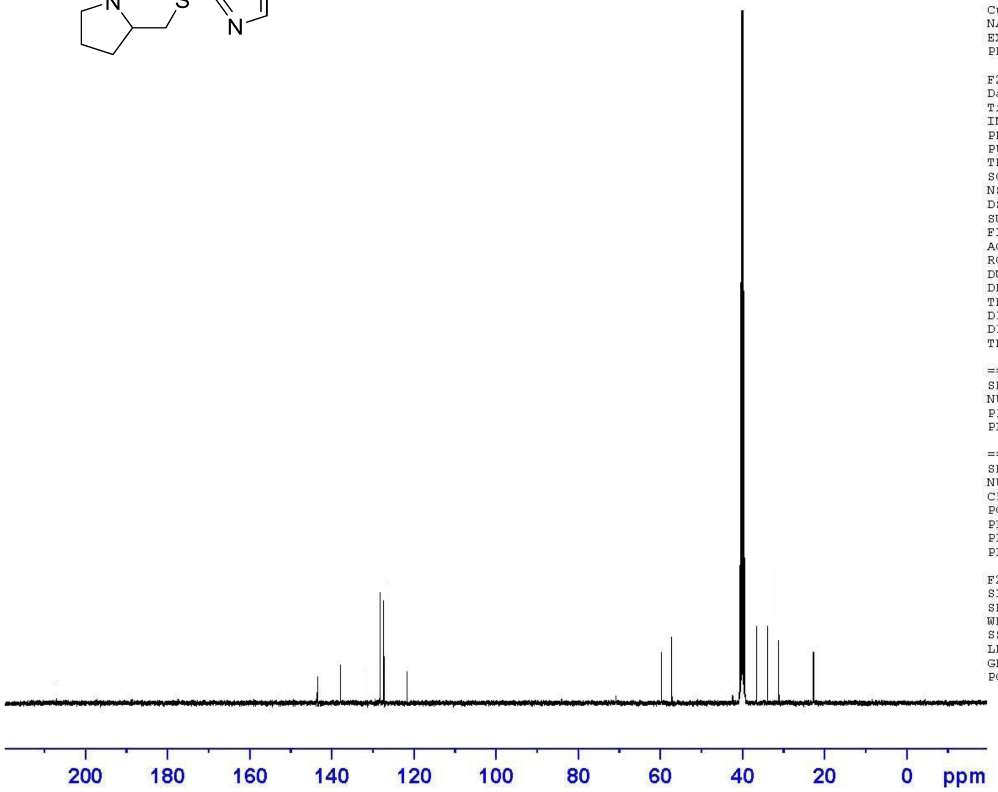


BRUKER

```
Current Data Parameters
NAME       PM-278_DMSO-
EXPNO                11
PROCNO                1

F2 - Acquisition Parameters
Date_          20140306
Time              19.37
INSTRUM           spect
PROBHD   5 mm PABBO BB/
PULPROG         zgpg30
TD               65536
SOLVENT           DMSO
NS                1024
DS                   4
SWH          24038.461 Hz
FIDRES        0.366798 Hz
AQ           1.3631488 sec
RG              199.98
DW              20.800 usec
DE                6.50 usec
TE               303.1 K
D1          2.00000000 sec
D11         0.03000000 sec
TD0                  1

======== CHANNEL f1 ========
SFO1       100.6228293 MHz
NUC1               13C
P1                9.75 usec
PLW1       75.00000000 W

======== CHANNEL f2 ========
SFO2       400.1316005 MHz
NUC2                1H
CPDPRG[2         waltz16
PCPD2            90.00 usec
PLW2       21.00000000 W
PLW12       0.22000000 W
PLW13       0.17010000 W

F2 - Processing parameters
SI                32768
SF         100.6127685 MHz
WDW                  EM
SSB       0
LB                 1.00 Hz
GB        0
PC                 1.40
```
